# Molecular Insights into Fungal Glycosylphosphatidylinositol Transamidase Complex

**DOI:** 10.1002/advs.202511340

**Published:** 2025-10-14

**Authors:** Zhengkang Hua, Xuyang Ding, Yanan Wu, Di Zhang, Xinlin Hu, Ping Yang, Jiameng Li, Yi Tan, Junbo Liu, Mingjie Zhang, Min Zhang, Xiaotian Liu, Hongjun Yu

**Affiliations:** ^1^ Department of Biochemistry and Molecular Biology, School of Basic Medicine, Tongji Medical College and State Key Laboratory for Diagnosis and Treatment of Severe Zoonotic Infectious Diseases and Hubei Key Laboratory of Natural Active Polysaccharides Huazhong University of Science and Technology Wuhan 430030 China; ^2^ Department of Pathogen Biology, School of Basic Medicine, Tongji Medical College and State Key Laboratory for Diagnosis and Treatment of Severe Zoonotic Infectious Diseases Huazhong University of Science and Technology Wuhan 430030 China; ^3^ Department of Neuroscience, School of Life Sciences Southern University of Science and Technology Shenzhen 518055 China; ^4^ Cell Architecture Research Center Huazhong University of Science and Technology Wuhan 430030 China

**Keywords:** antifungal drug, glycobiology, glycosylphosphatidylinositol, GPI anchor, GPI transamidase

## Abstract

The glycosylphosphatidylinositol (GPI) biosynthesis pathway is critical for antifungal drug development. As a key component of this pathway, GPI transamidase (GPIT) catalyzes the attachment of GPI anchors to proteins, a process essential for fungal cell wall integrity and virulence. Despite its biological significance, structural and mechanistic insights into fungal GPIT remain limited. Here, a series of cryo‐electron microscopy structures capturing distinct functional states of *Saccharomyces cerevisiae* GPIT is reported, including GPIT complexed with a GPI anchor, GPIT bound to a substrate‐mimetic peptide, and an unprecedented dimeric GPIT assembly. These structures reveal the conserved GPI anchor binding site formed by Gab1 and Gpi16, as well as a key protein substrate recognition site, Gpi16 Y550. Comparative structural analyses uncover fungal‐specific adaptations and the dynamic accommodation of catalytic subunit Gpi8. The dimeric GPIT structure exhibits a unique T‐shaped organization unexpectedly mediated by transmembrane helices of Gab1 and Gaa1, a configuration unlikely to form in the human counterpart. This study provides a molecular framework for understanding GPIT function and species‐specific divergences, providing a molecular basis for antifungal drug development.

## Graphical abstract

The glycosylphosphatidylinositol (GPI) biosynthesis pathway is critical for antifungal drug development. This study reports three cryo‐electron microscopy structures of fungal GPI transamidase (GPIT), captured in two substrate‐bound states and a novel dimeric assembly. Structural and functional characterizations elucidate the mechanisms underlying complex assembly, substrate recognition, and species‐specific features. These findings will facilitate drug discovery targeting fungal GPIT.

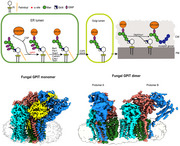

## 1 Introduction

Glycosylphosphatidylinositol (GPI), a universal eukaryotic anchor for cell surface proteins, plays a critical role in fungal biology by tethering key structural and virulence factors to the cell wall.^[^ ^1^, ^2^ ^]^ In fungi, including many prominent fungal pathogens, GPI‐anchored proteins (GPI‐APs) constitute the essential mannoprotein layer of the cell wall and include major virulence determinants, such as adhesins and invasins.^[^ ^3^, ^4^ ^]^ Given their indispensable roles in fungal adhesion, virulence, cell wall integrity, and survival, GPI biosynthesis has emerged as an attractive target for antimycotics development.^[^ ^5^, ^6^ ^]^ Manogepix, a first‐in‐class inhibitor that targets GPI inositol acylation, has progressed to Phase III clinical trials, underscoring the therapeutic potential of this pathway.^[^ ^7^ ^]^ Pioneering studies using *Saccharomyces cerevisiae (S. cerevisiae)* as a model system have been instrumental in identifying multiple lead compounds targeting key steps of GPI biosynthesis: inhibitors of GPI inositol acylation (e.g., BIQ, Gepinacin, G884, and G365),^[^ ^8^, ^9^, ^10^ ^]^ compounds disrupting ethanolamine phosphate transfer (e.g., YW3548 and M720),^[^ ^10^, ^11^ ^]^ and Jawsamycin that blocks the transfer of N‐acetylglucosamine to phosphatidylinositol.^[^ ^12^ ^]^

**Figure 1:**
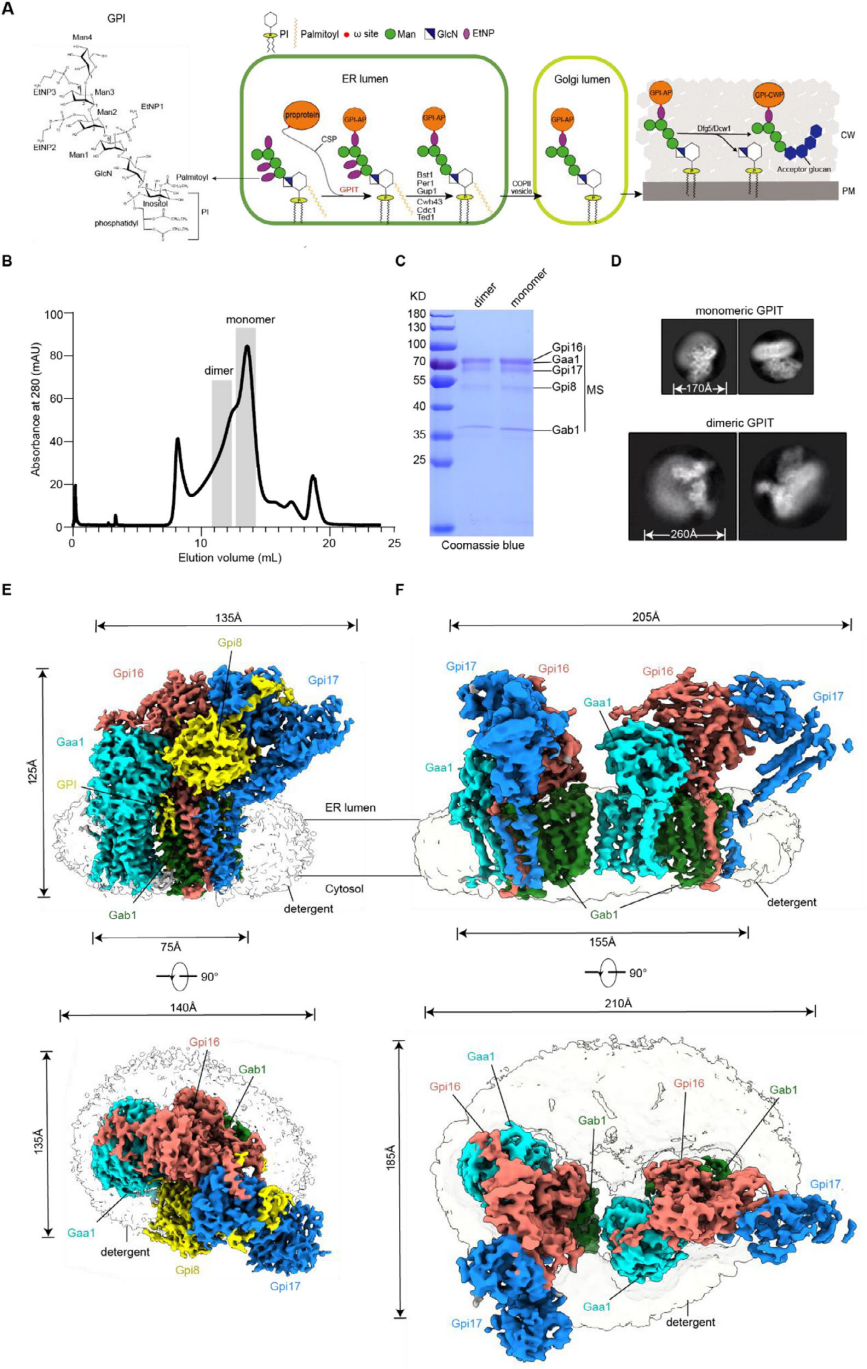
Cryo‐EM analysis of monomeric and dimeric GPIT. A) GPIT and the biosynthesis of GPI‐APs. GPIT catalyzes the formation of GPI‐APs via a transamidation reaction within the ER lumen. Specifically, GPIT cleaves the CSP of substrate proproteins at the ω‐site and attaches a GPI anchor via an amide bond to the newly exposed C‐terminal residue. After covalent attachment, the GPI anchor undergoes structural remodeling (e.g., lipid and glycan processing). The resulting mature GPI‐AP is then transported to the plasma membrane or extracellular space to participate in critical biological processes such as cell wall biogenesis. On the far left is depicted the canonical chemical structure of a GPI anchor, which comprises phosphatidylinositol (PI) lipid moiety, conserved glycan core (Manα1‐2Manα1‐6Manα1‐4GlcN), and multiple ethanolamine phosphate (EtNP) groups. B,C) Elution profile (B) and SDS‐PAGE analysis (C) of SEC (Superose 6 10/300 GL) purified GPIT complex. Grey boxes mark the fractions pooled for cryo‐EM sample preparation. The proteins were identified by mass spectrometry (MS) analysis as shown in Figure S1A (Supporting Information). D) Representative 2D images of the monomeric (top row) and dimeric (bottom row) GPIT. For each state, the top view (left) and side view (right) are displayed. E,F) Cryo‐EM maps of the GPIT complex in monomeric (E) and dimeric (F) states, with subunits individually segmented. The detergent disk (depicted in transparent grey) exhibits a significant size expansion in dimeric GPIT compared to the monomeric GPIT.

The GPI transamidase (GPIT) plays a crucial role in the GPI biosynthesis by facilitating GPI anchoring within the endoplasmic reticulum. The canonical GPI structure consists of a phosphatidylinositol (PI) lipid moiety covalently linked to a conserved glycan core (Manα1‐2Manα1‐6Manα1‐4GlcN), which have multiple phosphoethanolamine (EtNP) modifications (**Figure** **1**A). GPIT mediates the attachment of GPI molecules to nascent proteins bearing a C‐terminal signal sequence (CSP) (Figure 1A).^[^ ^3^, ^13^, ^14^ ^]^ In fungi, GPIT manifests as a membrane‐bound complex comprising five subunits: Gaa1, Gab1, Gpi8, Gpi16, and Gpi17, akin to GPAA1, PIGU, PIGK, PIGT, and PIGS in mammals.^[^ ^3^, ^15^ ^]^ While the abrogation of GPIT in mammals hampers embryogenesis and development,^[^ ^16^, ^17^, ^18^, ^19^, ^20^ ^]^ its indispensability for fungal growth and virulence is evident: *GPI8* is essential for *Candidaalbicans*, *Cryptococcusneoformans*, and *S. cerevisiae*;^[^ ^21^ ^]^ *GAA1* is essential for viability in both *Colletotrichum graminicola* and *S. cerevisiae*;^[^ ^22^ ^]^ *GPI16*, *GPI17*, and *GAB1* mutants in *S. cerevisiae* are inviable.^[^ ^22^, ^23^ ^]^ Furthermore, genetic disruption in GPIT subunits impaired GPI‐anchoring of cell wall proteins, resulting in cell wall abnormalities, altered drug responses, and attenuated pathogen virulence.^[^ ^22^, ^24^, ^25^ ^]^ These findings indicate GPIT as a potential therapeutic target, particularly given the growing pharmacological interest in GPI biosynthesis inhibition as an antifungal strategy.^[^ ^5^, ^6^ ^]^ It is, therefore, imperative to characterize fungal GPIT and to elucidate its structural and functional distinctions from its mammalian counterpart. While recent structural studies have advanced our understanding of human GPIT,^[^ ^26^, ^27^, ^28^ ^]^ a dearth of structural insights into fungal GPIT persists. A key unanswered question concerns the dimerization of fungal GPIT,^[^ ^29^, ^30^, ^31^, ^32^, ^33^, ^34^ ^]^ whose molecular basis remains a mystery.

Here we report cryo‐electron microscopy (cryo‐EM) structures of *S. cerevisiae* GPIT captured in multiple functional states, including complexes with a GPI anchor, a substrate‐mimetic peptide, as well as an unprecedented dimeric state. Integrated with structure‐guided functional characterizations, these results reveal the mechanisms governing fungal GPIT assembly, substrate recognition, and species‐specific features, providing a structural framework for antifungal drug development.

## 2 Results

### 2.1 Oligomeric States and Structure Determination of Fungal GPIT

To advance our understanding of the molecular mechanism of fungal GPIT, we focused on *S. cerevisiae* GPIT as our model system, leveraging its extensive functional characterizations while addressing the current gap in molecular insights.^[^ ^24^, ^35^ ^]^ To reconstitute the GPIT complex for structural studies, we co‐expressed all five components (Gaa1, Gab1, Gpi8, Gpi16, and Gpi17) and tagged the C‐terminus of Gpi8 with a triple FLAG tag. The resulting GPIT complex was purified using the detergent glycol‐diosgenin (GDN) via immunopurification method. Subsequent size‐exclusion chromatography (SEC) analysis revealed two major elution peaks: one corresponding to an ≈300 kDa species, and the other to a higher molecular weight species (≈700 kDa) (Figure 1B). Mass spectrometry (MS) analysis confirmed the presence of all known GPIT components in both peaks (Figure S1A, Supporting Information). The comparable subunit stoichiometry between these two peaks suggests that they differ only in oligomeric states (Figure 1C). Indeed, cryo‐EM analysis of GPIT in these two peaks allowed us to reconstruct the map of monomeric and dimeric GPIT at overall resolutions of 3.6 and 4.4 Å, respectively (Figure 1D–F; Figures S2–S4 and Table S1, Supporting Information). Notably, the dimeric structure represents the first structural visualization of the oligomeric states of fungal GPIT, corroborating previous observations of its dimerization.^[^ ^29^, ^30^, ^31^, ^32^, ^34^ ^]^

### 2.2 The Architecture of Monomeric GPIT

**Figure 2:**
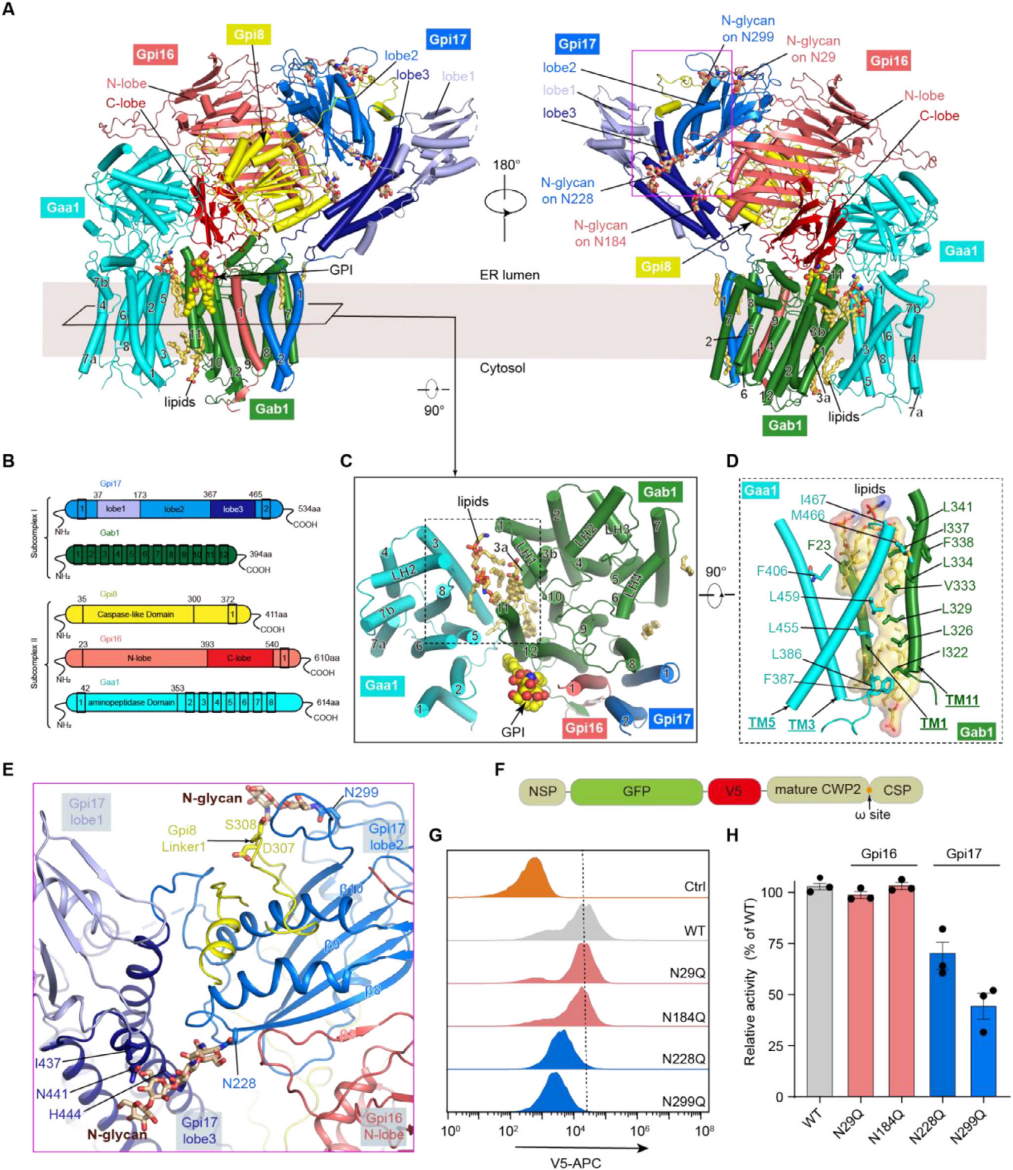
Cryo‐EM structure of monomeric GPIT. A) Cartoon representation of monomeric GPIT structure, shown in two orientations (left, front; right, back view). Individual subunits are color‐coded as labelled, while subdomains of Gpi16 and Gpi17 are distinguished by specific red‐ and blue‐based color tones, respectively. The bound GPI (yellow sphere), N‐glycans (wheat sticks), and ordered lipids (yellow sticks) are labelled. B) Schematic diagrams of the individual subunits of the GPIT complex. Subunits are colored as those in (A), with domains labelled and TM (transmembrane) helices depicted as numbered rectangular boxes. C) ER luminal view of the GPIT TM helices (denoted by numbers). D) Close‐up view (rotated by 90 degrees) of the Gaa1‐Gab1 interface outlined by the dashed box in (C). Residues mediating subunit interactions are represented as sticks. Enriched lipids stabilizing this interface are depicted in sticks with transparent surface rendering. E) Glycan‐mediated inter‐subunit (N‐glycan on Gpi17 N299) and intra‐subunit (N‐glycan on Gpi17 N228) interactions, as outlined by the pink box in (A, right). F) Schematic of the engineered construct designed for GPIT functional assessment. The CWP2 is utilized as a GPI‐anchored reporter, with GFP and V5 epitope incorporated downstream of its N‐terminal signal peptide (NSP). G) In vivo functional analysis of glycosylation site mutations, with representative FACS (fluorescence‐activated cell sorting) results shown. Strains carrying indicated chromosomal mutations were transformed with a plasmid expressing the GFP‐V5‐CWP2 construct in (F). The surface expression analysis of CWP2 was quantified by FACS using iFluor 647‐conjugated V5 tag antibody (see methods). The gating strategy is shown in Figure S7A (Supporting Information). The control (orange) represents the V5‐staining (iFluor 647) background of yeast cells without the transformation of GFP‐V5‐CWP2 construct. H) Quantitative data (% of wild‐type) of FACS‐based analysis in (G), with the graph representing the mean ± s.e.m. of three independent measurements.

The density map of monomeric GPIT is of sufficient quality to allow the building of most residues (Figures S2 and S3, Supporting Information). The resulting atomic model comprises a single copy of each subunit–Gaa1, Gab1, Gpi8, Gpi16, and Gpi17–spanning dimensions of ≈125 Å × 135 Å × 75 Å (Figure 1E and **Figure** **2**A). The architecture features a transmembrane (TM) domain and a prominent luminal entity, with no discernible cytoplasmic regions. The luminal entity is formed by the soluble domains of Gaa1, Gpi8, Gpi16, and Gpi17, while the TM domain is assembled from 23 TM helices contributed by Gaa1 (eight helices), Gab1 (twelve helices), Gpi16 (one helix), and Gpi17 (two helices) (Figure 2B,C).

The five GPIT subunits are organized into two distinct subcomplexes: Subcomplex I (Gab1 and Gpi17) and Subcomplex II (Gaa1, Gpi8, and Gpi16) (Figure 2A,B), consistent with previous studies identifying similar architectural divisions.^[^ ^23^, ^29^, ^34^ ^]^ In Subcomplex I, Gab1, an integral membrane protein, is embedded in the membrane via its twelve TM helices. Gpi17 features two transmembrane helices (TM1 and TM2) at its N‐ and C‐termini, which encircle Gab1's TM8 to anchor Gpi17 to the membrane (Figure 2C). The luminal domain of Gpi17 is elongated and organized into three subdomains (lobe 1, lobe 2, and lobe 3), forming a structural scaffold to contact Subcomplex II for holocomplex assembly (Figure 2A,B). Subcomplex II is maintained by interactions among the soluble domains of its constituent subunits: Gaa1, Gpi16, and Gpi8 (Figure 2A,B). This arrangement is consistent with the recent reconstitution of a minimal transamidase complex comprising soluble truncated domains of these three subunits.^[^ ^34^ ^]^ Gpi16 contains a single TM helix and a luminal domain composed of two β‐strand‐rich subdomains (N‐lobe and C‐lobe). Gaa1 is anchored to membrane through its eight TM helices while its luminal domain, resembling zinc‐dependent aminopeptidases,^[^ ^36^ ^]^ interacts with the N‐ and C‐lobes of Gpi16 in a shape‐complementary manner. Gpi8, the catalytic subunit, adopts a caspase‐like fold and forms extensive interactions with both Gaa1 and Gpi16 simultaneously, further stabilizing the Subcomplex II (Figure 2A).

The assembly of GPIT holocomplex relies on extensive interactions between Subcomplexes I and II, spanning both the luminal and membrane regions. On the luminal side, lobe 2 and lobe 3 of Gpi17 interact extensively with the N‐lobe of Gpi16 and the luminal domain of Gpi8, while the C‐lobe of Gpi16 rests right above Gab1 (Figure 2A). In contrast, the membrane‐side interfaces between Subcomplex I and II are relatively loose, with limited protein‐protein interactions. On one side, the sole TM helix of Gpi16 fills snugly between Gab1 TM12 and Gpi17 TM2, forming a stable three‐helix sheet‐like arrangement (Figure 2C). On the other side, Gaa1 loosely packs against Gab1 through interactions primarily between Gaa1 TM3/TM5 and Gab1 TM1/TM11, leaving a sizeable gap between them (Figure 2C). Notably, this gap is stabilized by eight ordered lipids (four in the luminal leaflet and four in the cytosolic leaflet) identified at the Gaa1‐Gab1 interface, which contribute to maintaining the integrity of the complex (Figure 2D; Figure S3B, Supporting Information).

Although the overall architecture of the GPIT complex is evolutionarily conserved between yeast (yGPIT) and human (hGPIT) orthologs, our structural analysis reveals substantial divergences in inter‐subunit interfaces in the context of only moderate sequence conservation between these species (Table S2, Supporting Information). Given the remarkably huge interaction surface, we have focused on a subset of interface regions that exhibit fungal‐specific conservation patterns, which may have important functional implications (Figure S5, Supporting Information). At the yGaa1‐yGpi16 interface, the L_α5‐β9_ loop of yGpi16 is extended by 11 residues, forming a clamp‐like element that pins yGaa1 (Figure S5B, Supporting Information). In the cytoplasmic‐facing juxtamembrane region, both yGpi16 and yGab1 feature extra short β‐strands, interlocking to form stronger packing interface between yGpi16 TM1 and yGab1 TM12 (Figure S5C, Supporting Information). At the yGpi16‐yGpi17 interface, the U‐turn loop (L_β2‐β3_) of yGpi16 is more extended in fungi, allowing stronger interactions with the neighboring β8 strand of yGpi17 (Figure S5E, Supporting Information). The L_α4‐β5_ of yGaa1 loop is 9‐residue shorter and lacks an N‐glycosylation site present in hGPAA1 (N203), leading to a different engaging mode with β14 strand of yGpi16 (Figure S5F, Supporting Information). Notably, the catalytic subunit yGpi8 also exhibits unique features in yGPIT, as discussed below.

### 2.3 Glycosylation of Yeast GPIT

Previous studies suggested that the yeast GPIT complex contains N‐glycosylation on Gpi16 and Gpi17.^[^ ^29^, ^37^ ^]^ Our structure of yGPIT was of sufficient resolution to visualize the glycans and identify four glycosylation sites: N29 and N184 of Gpi16, N228 and N299 of Gpi17 (Figure 2A; Figure S3C, Supporting Information). Although Gpi17 N228 is conserved in its human counterpart (hPIGS N267) (Figure S6, Supporting Information), we observed density covering four glycan moieties (GlcNAc_2_Man_2_) at Gpi17 N228, which is longer than the single saccharide found at hPIGS N267 (Figure S3C, Supporting Information). This more ordered glycan, located in lobe 2 of Gpi17, interacts with residues (I437, N441, and H444) in lobe 3 of Gpi17, suggesting its exclusive role in inter‐domain stabilization in yGPIT. Of the remaining glycans (at N29 and N184 of Gpi16, N299 of Gpi17), the N299 glycan of Gpi17 participates in interactions with proteins, packing against the C‐terminal linker of Gpi8 (Figure 2E).

Given the lack of a robust method to assess the GPIT activity in yeast or any other fungi, we developed a fluorescence‐activated cell sorting (FACS) assay to evaluate the functional relevance of these structural observations (Figure 2F; Figure S7A, Supporting Information; see Experimental Section for details). In this assay, we targeted CWP2, a GPI‐anchored protein, by constructing a chimera that fuses CWP2 with GFP and a V5 linker (Figure 2F). Cell surface levels of this chimeric CWP2 were monitored by flow cytometry in yeast cells carrying chromosomal GPIT mutations. Consistent with our structural findings, Gpi17 N228Q and N299Q mutations to remove N‐glycosylation significantly impaired GPIT activity compared to the wild‐type, while Gpi16 N29Q and N184Q mutations had little effect on GPIT activity (Figure 2G,H). Taken together, these functional and structural studies indicate the role of the glycans at Gpi17 N228 and N299 in complex assembly and stability.

### 2.4 Dynamic Accommodation of Catalytic Subunit Gpi8 in Yeast GPIT Complex

**Figure 3:**
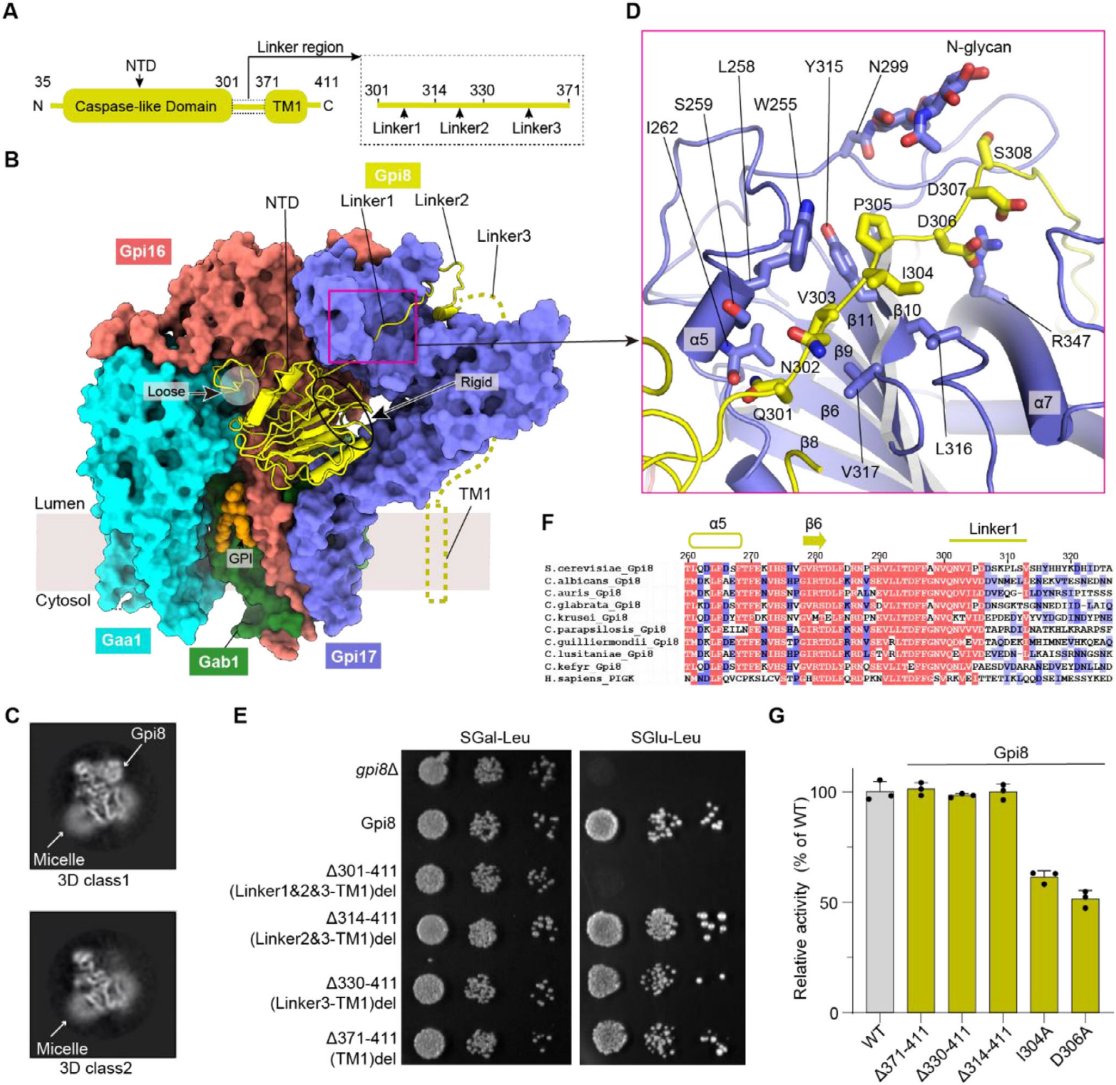
The anchoring of Gpi8 in the yGPIT complex. A) Domain architecture of Gpi8, comprising an N‐terminal domain (NTD) in the core region, an extended Linker region (Linker1‐3), and a predicted transmembrane helix (TM1). B) The binding mode of Gpi8. The Gpi8 NTD, adopting a Caspase‐like fold, engages in both dynamic and rigid interactions with neighboring subunits (see Figure S8, Supporting Information for details). Downstream of NTD, Gpi8 Linker1 wraps around Gpi17 lobe2, while Linker2 terminates in a cleft between Gpi17 lobe1 and lobe2. The disordered Linker3 and TM1 are depicted schematically. C) 3D classification analysis reveals two distinct states, with 3D class 2 exhibiting a notable absence of Gpi8 density compared to 3D Class 1. Slice‐views of both 3D classes are shown. D) Close‐up view of the interactions between Gpi8 Linker1 and Gpi17, corresponding to the region highlighted by the pink box in (B). E) Growth complementation of *BY4742‐HIS‐GAL‐GPI8* conditional lethal strains with empty plasmid (*gpi8*Δ) or plasmids carrying either *GPI8* wild‐type (Gpi8) or *GPI8* variants. The transformed cells were grown for 72 h at 30 °C on Yeast Synthetic Drop‐out Medium lacking leucine, supplemented with either 4% galactose (SGal‐Leu; left rank) or 2% glucose (SGlu‐Leu; right rank). The test was repeated three times with similar results. F) Multiple sequence alignment of Gpi8 Linker1 across human and fungal species, including S. cerevisiae and fungal pathogens (*C. albicans, C. auris, C. glabrata, C. krusei, C. parapsilosis, C. guilliermondii, C. lusitaniae, and C. kefyr*). G) In vivo functional analysis of Gpi8 mutations, showing quantitative FACS‐based assay results as a percentage of wild‐type activity. The graph represents the mean ± s.e.m. of three independent measurements. See Figure S7B (Supporting Information) and methods for more details. Notably, during the alanine scanning mutagenesis analysis of Linker1, only I304A and D306A could be successfully introduced into the *GPI8* chromosomal locus, consistent with the lethal phenotype of Gpi8 Linker1 deletion (E).

The structure of yGPIT reveals notable differences from hGPIT, particularly in the organization and dynamics of Gpi8. Structurally, Gpi8 contains an N‐terminal caspase‐like domain (NTD; residues 35‐300) characterized by a six‐stranded β‐sheet flanked by two pairs of parallel alpha‐helices (Figure 2B and **Figure** **3**A,B). Compared to its counterpart PIGK in hGPIT, Gpi8 NTD in yGPIT exhibits pronounced binding flexibility, which is unevenly distributed across its structure (Figure S8A,B, Supporting Information). One side of the Gpi8 NTD is stably anchored by a concave interface formed by Gpi16 and Gpi17, creating a rigid binding surface (Figure 3B; Figure S8A, Supporting Information). In contrast, the opposing site of the Gpi8 NTD is markedly flexible, likely due to insufficient stabilization (Figure S8A, Supporting Information). The flexible region includes a long loop of Gpi8 (residues 81‐108; with only partial main‐chain tracing) that extends toward Gpi16 (Figure 3B). In hGPIT, this interface is stabilized by an inter‐subunit disulfide bond between PIGK C92 and PIGT C182, corresponding to yGpi8 C85 and yGpi16 C202.^[^ ^26^, ^27^ ^]^ However, our structure indicates that such a disulfide bond is unlikely in yGPIT as the helix containing yGpi16 C202 is retracted (Figure S8E,F, Supporting Information). This is consistent with previous biochemical characterization,^[^ ^31^ ^]^ underlying the increased binding flexibility at this interface. Additionally, 3D classification identified an unexpected particle population lacking yGpi8 density (Figure 3C), even though the GPIT complex was purified using an affinity tag on the yGpi8 subunit. This finding reinforces the dynamic nature of yGpi8 binding within yGPIT complex, likely the last subunit to incorporate during complex assembly as inferred from previous studies.^[^ ^38^ ^]^

Following its NTD, Gpi8 interacts with Gpi17 through an extended linker region (Figure 3A,B; Figure S8A, Supporting Information). This linker adopts a dual‐anchoring configuration: its N‐terminal segment (Linker1; residues 301‐313) wraps around Gpi17 lobe2, while its C‐terminal segment (Linker2; residues 314‐329) terminates in a cleft between Gpi17 lobe1 and lobe2 (Figure 3B,D). Systematic truncation analysis demonstrated that Linker1 is essential for Gpi8‐dependent cell viability, whereas Linker2 is dispensable (Figure 3E). Notably, this Gpi8 Linker1 region is more conserved across yeast and fungal pathogens but shows less conservation with its human counterpart, consistent with the reorganized binding sites for Linker1 region between yeast and human (Figure 3F; Figure S8A–D, Supporting Information). Structural analysis uncovered that the more functionally critical Linker1 occupies the cleft between α5 and β10, forming interactions of mixed nature (Figure 3D): hydrophobic interactions include Gpi8 V303 engaging Gpi17 L258 and V317, Gpi8 I304 engaging Gpi17 W255 and L316, and Gpi8 P305 engaging Gpi17 Y315; polar interactions include Gpi8 D306 with Gpi17 R347, Gpi8 N302 with Gpi17 S259, and Gpi8 Q301 with the main chain of Gpi17 I262. This extensive network of interactions mediated by Linker1 stably anchors the catalytic subunit Gpi8 in an optimal position within the complex, which is critical for its catalytic function (Figure 3B). Indeed, in our subsequent alanine scanning mutagenesis analysis of Linker1, only the I304A and D306A chromosomal mutants could be generated, consistent with the lethal phenotype of the deletion of Gpi8 Linker1 (Figure 3E). As expected, both mutants exhibited significantly impaired GPIT activity in FACS‐based functional assays, demonstrating Linker1's critical role in GPIT function (Figure 3G; Figure S7B, Supporting Information).

Downstream of the Gpi8 Linker1&2 region, there is a predicted long loop (Linker3; residues 330‐370) and a transmembrane helix (TMH; residues 371‐411) that remained unresolved in our structure (Figure 3B), consistent with the dynamic binding mode of Gpi8 in yGPIT. In contrast, in hGPIT, the C‐terminal loop and TMH of hPIGK (the human equivalent of yGpi8) contribute to the stable anchoring of hPIGK.^[^ ^26^, ^27^ ^]^ Notably, we found that yGpi8 Linker3 and TMH are dispensable (Figure 3E), as their truncations did not impair yGPIT activity in vivo (Figure 3G; Figure S7B, Supporting Information). Moreover, we successfully purified the full complex in both monomer and dimer states even upon deletion of the yGpi8 TMH (Figure S8G,H, Supporting Information). These results are consistent with previous studies that reported soluble forms of Gpi8 lacking the C‐terminal TMH in certain eukaryotes, such as *Trypanosoma brucei, Caenorhabditis elegans, and Drosophila melanogaster*.^[^ ^39^, ^40^, ^41^ ^]^

### 2.5 The Binding of GPI Ligand

**Figure 4:**
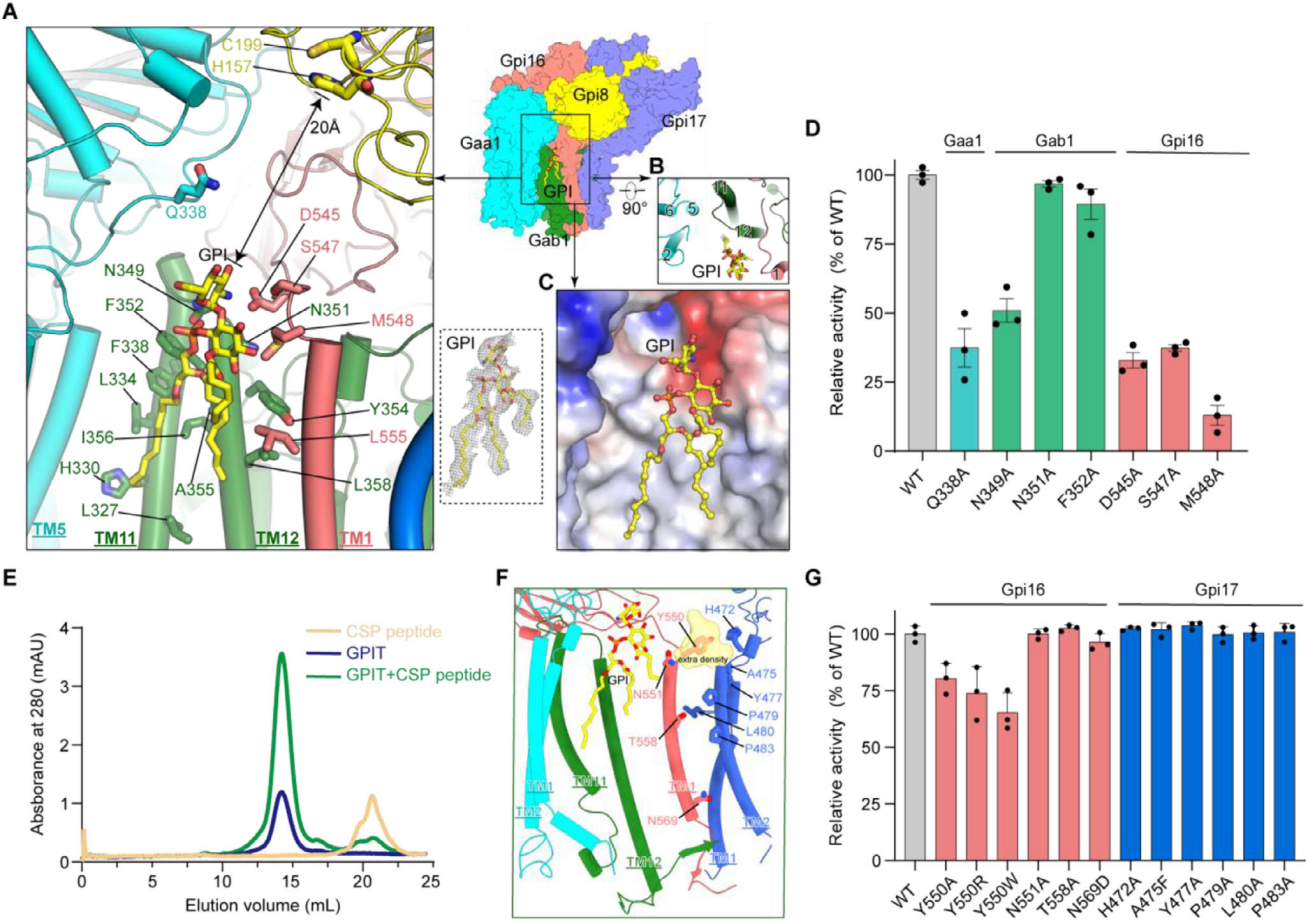
Substrate binding in yGPIT. A) Binding of the GPI ligand, viewed parallel to the membrane. Key residues from Gab1 (green sticks) and Gpi16 (red sticks) collectively form a binding site to accommodate the GPI molecule (yellow stick). The His‐Cys catalytic dyad (H157 and C199) of Gpi8 is positioned ≈20 Å above the bound GPI. The inset (dashed box) shows the modelled GPI molecule (yellow stick) superimposed with the electron microscopy density (grey mesh). B) Luminal view of the GPI binding site, formed by TM11‐12 of Gab1 and TM1 of Gpi16. C) Electrostatic surface potential of the GPI binding site, with blue color for positive charges, white for neutral, red for negative. The bound GPI is shown in ball‐and‐stick representation. D) In vivo functional analysis of GPI‐binding mutations, showing quantitative FACS‐based assay results as a percentage of wild‐type activity. The graph represents the mean ± s.e.m. of three independent measurements. See Figure S7C (Supporting Information) and methods for more details. E) SEC (Superose 6 10/300 GL) of purified GPIT in the presence and absence of CSP of MKC7. F) Extra density (semi‐transparent yellow) observed in the GPIT‐CSP peptide reconstruction suggests a peptide substrate binding site. Positioned adjacent to the bound GPI, this density directly contacts Gpi16 Y550 and Gpi17 H472 and is absent in GPIT map without CSP peptide incubation (Figure S9F, Supporting Information). Conserved yGPIT residues proposed for CSP binding in human GPIT complex (PDB ID: 8IMY), are shown as sticks. G) In vivo functional analysis of CSP‐binding mutations, showing quantitative FACS‐based assay results as a percentage of wild‐type activity. The graph represents the mean ± s.e.m. of three independent measurements. See Figure S7D (Supporting Information) and methods for more details.

GPIT‐mediated protein transamidation relies on the evolutionarily conserved residues of Gpi8 (H157 and C199), which have been identified as key catalytic sites in yGPIT based on their analogy to cysteine proteases.^[^ ^38^ ^]^ Our structure shows that Gpi8 positions its His‐Cys catalytic dyad (H157 and C199) facing the membrane (**Figure** **4**A). Approximately 20 Å below this catalytic site, a prominent non‐protein density is observed within a V‐shaped hydrophobic cavity, lined by TM5 of Gaa1, TM11‐12 of Gab1, and the sole TM of Gpi16 (Figure 4A,B). The shape of this density strongly suggests that it accommodates a GPI molecule, specifically one bearing a palmitoylated phosphatidylinositol (PI) moiety and a glucosamine (GlcN) moiety (Figure 4A, inset).^[^ ^42^ ^]^ The density does not clearly define the positions of the mannose and phosphoethanolamine (EtNP) moieties, indicating either their dynamic connection. Alternatively, the mannose residues may have been partially trimmed by mannosidases during purification,^[^ ^43^ ^]^ as previous studies of human GPIT structures have also identified bound GPI molecules with varied glycan lengths.^[^ ^26^, ^27^ ^]^

The presence of an endogenous ligand at this site underscores its high affinity and selectivity for GPI. The headgroup of the GPI molecule (comprising the GlcN moiety, inositol ring, and phosphate group) is positioned at the luminal face, stabilized via polar interactions with N349 and N351 of Gab1, as well as S547 and D545 of Gpi16, alongside hydrophobic packing with M548 of Gpi16 (Figure 4A,C). The three acyl chains of GPI extend into the luminal membrane leaflet and engage in extensive van der Waals interactions with surrounding hydrophobic residues, including L327, H330, L334, and L338 on Gab1 TM11; F352, Y354, A355, I356, and L358 on Gab1 TM12; L555 on Gpi16 TM1 (Figure 4A,C). Additionally, the highly conserved Gaa1 Q338 extends over the GPI anchor, potentially contacting mannose/EtNP moieties that could not be modeled due to the lack of density (Figure 4A,C). To probe the functional relevance of residues lining the GPI binding site, we introduced chromosomal mutations and assessed in vivo GPIT activity using our FACS‐based assay. Alanine substitutions of D545, S547, and M548 of Gpi16, Gab1 N349 as well as Gaa1 Q338, resulted in a substantial (>50%) loss of activity, whereas N351A and F352A mutations of Gab1 had no discernible effect (Figure 4A,D; Figure S7C, Supporting Information).

### 2.6 The Binding of Acceptor C‐Terminal Signal Peptide

GPIT catalyzes the attachment of GPI to the C‐terminus of proprotein by replacing its CSP.^[^ ^15^ ^]^ To investigate the molecular basis of CSP binding of yGPIT, we synthesized the CSP peptide (residues 564‐596) of MKC7, a GPI‐anchored aspartyl protease involved in cell wall maintenance.^[^ ^44^ ^]^ SEC analysis confirmed the binding of this CSP‐mimetic peptide to the purified GPIT complex carrying the catalytically inactive Gpi8 C199S mutation (Figure 4E).^[^ ^38^ ^]^ We subsequently determined the structure of the CSP‐bound GPIT complex at an overall resolution of 3.3 Å (Figure S9A–E, Supporting Information). The core GPIT structure remained unchanged, but additional electron density appeared near the GPI molecule only in the presence of CSP peptide, with no equivalent density observed in the untreated control (Figure S9F, Supporting Information). This additional density is positioned between the luminal ends of Gpi16 TM1 and Gpi17 TM2, and is stabilized by Gpi16 Y550 and Gpi17 H472, suggesting a potential CSP binding site (Figure 4F). This structural arrangement is evolutionarily conserved, as Y526 in human PIGT (the orthologous residue to yeast Gpi16 Y550) similarly contacts the CSP substrate, though its functional significance remains uncharacterized.^[^ ^28^ ^]^ Our characterization revealed that while the Gpi17 H472A mutation had no discernible effect, substitutions at the conserved Gpi16 Y550 (to alanine, arginine, or tryptophan) consistently reduced GPIT activity to 70%–80% of wild‐type levels (Figure 4G; Figure S7D, Supporting Information), underscoring the importance of Gpi16 Y550 in CSP recognition.

Furthermore, CSPs typically feature a hydrophobic region of 15–20 residues.^[^ ^15^ ^]^ However, the corresponding density is not visible in our structural map, suggesting conformational flexibility. Structural analysis of human GPIT indicates that the CSP hydrophobic region of ProULBP2 interacts with PIGT TM1 and PIGS TM2—the equivalents of yeast Gpi16 TM1 and Gpi17 TM2, respectively (Figure S9G, Supporting Information). To investigate whether this recognition mechanism is conserved in yGPIT, we performed site‐directed mutagenesis of key conserved residues proposed for CSP binding in human GPIT (residues N551, T558, and N569 on yGpi16 TM1; residues A475, Y477, P479, L480, and P483 on yGpi17 TM2) and systematically evaluated their functional impacts. Surprisingly, unlike their counterparts in human GPIT, none of these mutations produced discernible phenotypic effects (Figure 4G; Figures S7D and S9H, Supporting Information). Furthermore, the autoinhibitory loop, which has been proposed to regulate substrate binding in human GPIT,^[^ ^28^ ^]^ shows lower homology with the corresponding region in yGPIT (Figure S9I, Supporting Information). These results implicate divergence in the recognition of the CSP hydrophobic region between yeast and humans,^[^ ^45^ ^]^ highlighting an important point for further investigation.

### 2.7 The Architecture of Dimeric GPIT

Previous studies have suggested the higher‐order oligomeric state of GPIT, though its precise assembly remains elusive.^[^ ^29^, ^30^, ^31^, ^32^, ^33^, ^34^ ^]^ Our cryo‐EM structure of dimeric yGPIT provides unprecedented insights into its oligomeric assembly. The high‐quality density map allowed unambiguous docking of two monomeric yGPIT models, revealing well‐defined secondary structural features and transmembrane helices (Figure 1F; Figure S4, Supporting Information). Intriguingly, while our biochemical analysis confirmed the presence of all subunits in the affinity‐purified complex (via the affinity tag on Gpi8; Figure 1A,B), Gpi8 was noticeably absent from the dimer reconstruction. This finding, consistent with the observed flexibility of Gpi8 in the monomeric structure (Figure 3; Figure S8A, Supporting Information), reinforces the dynamic accommodation of Gpi8 within the yeast GPIT complex.

**Figure 5:**
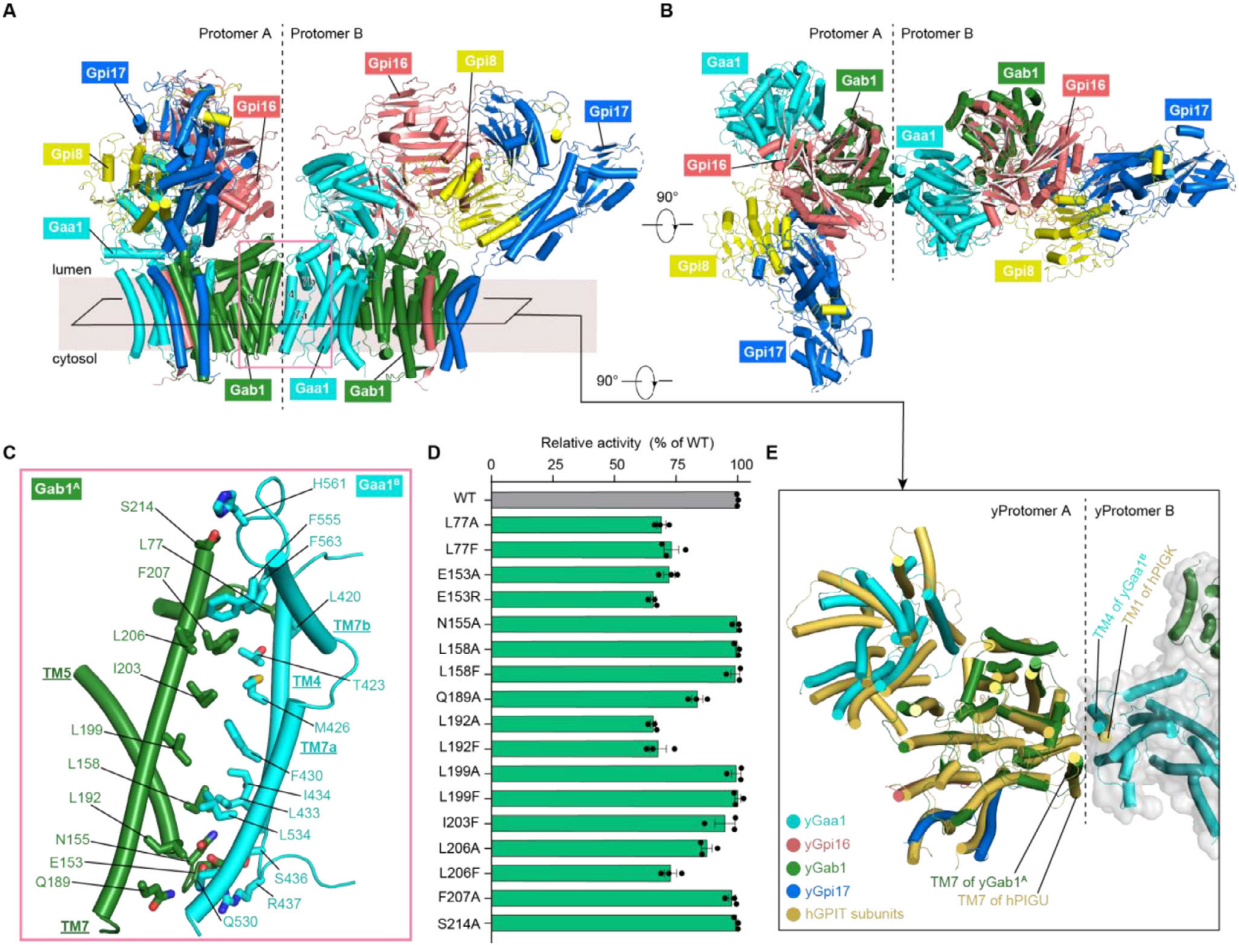
Architecture of dimeric GPIT. A,B) Two orthogonal views of the dimeric GPIT complex in cartoon display. A) View parallel to the membrane. B) View from the luminal side. Subunits from protomer A and B are colored consistently with Figure 2A. Although Gpi8 is disordered in the dimeric complex and could not be resolved structurally, it has been hypothetically modeled in these panels to illustrate the putative intact assembly. C) Close‐up view of the dimeric interface, outlined by the pink box in (A). This interface is formed between the Gab1 subunit of protomer A and the Gaa1 subunit of protomer B, with potential interacting residues shown in stick representation. D) In vivo functional analysis of dimer interface mutations in Gab1, showing quantitative FACS‐based assay results as a percentage of wild‐type activity. The graph represents the mean ± s.e.m. of three independent measurements. See Figure S7E (Supporting Information) and methods for more details. E) Structural superimposition of monomeric human GPIT (PDB: 7WLD; all subunits in light orange) onto protomer A of dimeric yGPIT (subunits colored as indicated). Only the transmembrane domains of the aligned structures are shown. Notably, this analysis reveals that TM1 of human PIGK aligns with TM4 of yGaa1 from protomer B in yGPIT dimer, suggesting the dimerization mode in yGPIT is unlikely in human GPIT.

The dimeric GPIT is embedded in an enlarged micelle disk (≈210 Å × 185 Å), markedly larger than that of monomeric GPIT (≈140 Å × 135 Å) (Figure 1D–F). The overall structure of the GPIT dimer adopts a T‐shape, where two protomers interact solely through their transmembrane regions via Gab1 and Gaa1 from each protomer (**Figure** **5**A,B). Although Gpi8 is disordered in the dimeric complex and could not be resolved structurally (Figure 1F), it was modeled based on the GPIT monomer to illustrate the putative intact assembly (Figure 5A,B). Notably, this arrangement orients the two Gpi8 binding sites in opposite directions, rendering them sterically inaccessible for dimerization through the caspase‐like Gpi8 subunit. This is contrary to previous proposals,^[^ ^30^, ^31^, ^33^ ^]^ indicating a novel mode of GPIT oligomerization.

Detailed analysis of the structure revealed the specific inter‐protomer interfaces for dimerization, with Gab1 TM5/TM7 in protomer A packing against Gaa1 TM4/TM7 in protomer B (Figure 5A). The interface is primarily mediated through van der Waals contacts among hydrophobic residues, including L77, L158, L192, L199, I203, L206, and F207 of Gab1, and L420, T423, M426, F430, L433, I434, L534, and F563 of Gaa1 (Figure 5C). In addition, potential polar interactions also contribute to dimer stabilization, such as S214 of Gab1 with H561 of Gaa1 at the luminal face, and E153 and N155 of Gab1 with S436 of Gaa1, as well as Q189 of Gab1 with Q530 of Gaa1 at the cytosolic face (Figure 5C). To functionally validate these structural observations, we performed systematic scanning mutagenesis targeting the dimer interface residues in Gab1. Subsequent FACS‐based analysis revealed that mutations such as L77A/F, E153A/R, Q189A, L192A/F, and L206A/F resulted in a significant reduction in GPIT activity (Figure 5D; Figure S7E, Supporting Information), underscoring the critical role of the dimerization in maintaining GPIT function. Moreover, we purified the variant complexes with these functionally impactful mutations (L77F, E153R, Q189A, L192F, L206F), and analyzed their oligomerization states using SEC (Figure S10A, Supporting Information). While retaining the subunit stoichiometry comparable to that of the wild‐type complex, these variants shifted the oligomeric equilibrium toward the monomeric form to varying degrees (Figure S10A,B, Supporting Information). Notably, the L77F mutation resulted in the most pronounced disruption, with the monomeric species becoming predominant. These findings provide biochemical support for the functional importance of the dimer interface identified through our structural and in vivo functional analyses.

Strikingly, structural superposition of the human monomeric GPIT complex with protomer A of the dimeric yGPIT reveals that the sole transmembrane helix of human PIGK aligns closely with Gaa1 TM4 from protomer B, thereby preventing dimer formation in hGPIT (Figure 5E). This contrasts sharply with the yeast system, where the corresponding Gpi8 TMH exhibits significant conformational flexibility (Figure 3), a feature that likely facilitates yGPIT dimerization. Supporting this model, deletion of the Gpi8 TMH in yeast neither impaired GPIT activity nor prevented dimer complex purification (Figure 3E,G; Figure S8G,H, Supporting Information). These findings uncover a fundamental divergence in GPIT oligomerization mechanisms between species, which warrants further investigation.

## 3 Discussion

In this study, we present a series of structural snapshots capturing distinct functional states of the fungal GPIT complex, including GPIT in complex with a GPI anchor, GPIT bound to a substrate‐mimetic peptide, and a dimeric GPIT assembly. Combined with functional characterization, these structures provide critical insights into the molecular mechanisms of GPIT and bridge a gap in our understanding of its role in fungal GPI biosynthesis.

Our study reveals the precise composition of the binding site for GPI anchor. It is formed by a combination of polar and hydrophobic residues contributed by Gab1 and Gpi16 and it represents the most evolutionarily conserved region of the GPIT complex, emphasizing its key role in enzymatic function (Figure 4A–C; Figure S6, Supporting Information). Regarding protein substrate recognition, a previous study emphasized the importance of the hPIGT (the human counterpart of yGpi16) transmembrane helix in mammalian systems;^[^ ^28^ ^]^ however, our data indicate that, at least in yeast GPIT, a previously uncharacterized residue (yGpi16 Y550) upstream of this TM helix plays a more critical role in substrate engagement (Figure 4F,G; Figure S9G, Supporting Information). Although the CSP was designed to include the linker region encompassing the ω cleavage site, this segment was not resolved in the cryo‐EM density within the Gpi8 active site, likely due to its intrinsic flexibility (Figure S9F,G, Supporting Information). Future studies using substrates with higher binding affinity may help stabilize this region and allow detailed visualization of its interactions with the catalytic site. Notably, while prior studies have emphasized the dimeric functional assembly of the GPIT complex,^[^ ^29^, ^30^, ^31^, ^32^, ^33^, ^34^ ^]^ our work provides the first structural elucidation of this assembly state. Unexpectedly, our structure reveals a previously unrecognized dimerization interface mediated by transmembrane helix interactions rather than the anticipated Gpi8‐Gpi8 interaction interface,^[^ ^30^, ^31^, ^33^ ^]^ suggesting a novel mode for higher‐order complex formation (Figure 5A–C). Further studies are required to develop a thorough understanding of the in vivo functional mechanism of this oligomeric organization.

Our GPIT structures reveal conserved structural features and notable divergences from the human complex. We identify fungal‐specific inter‐subunit interface adaptations and unique protein‐stabilizing glycans different from the human GPIT (Figure 2E; Figure S5, Supporting Information). Moreover, the anchoring mode of the catalytic subunit yGpi8 exhibits striking differences: whereas human PIGK is stably positioned within the complex,^[^ ^26^, ^27^ ^]^ our data reveal that yGpi8 exhibits substantial dynamic binding features (Figure S8A,B, Supporting Information), and we captured a structural state in which yGpi8 is entirely absent (Figure 3C). Additionally, we identified a loop, yGpi8 Linker1, that is highly conserved in fungi and plays a key role in anchoring yGpi8 in place (Figure 3D–G). Importantly, our findings suggest that in human GPIT, the binding of the C‐terminal transmembrane helix of hPIGK sterically hinders the dimerization mode observed in fungal GPIT (Figure 5E), indicating a species‐specific quaternary organization that may contribute to functional divergence between fungal and human GPIT.

In summary, our study presents a comprehensive structural and functional characterization of the fungal GPIT complex, shedding light on its molecular architecture, substrate interactions, and species‐specific features. These findings not only advance our understanding of GPIT evolution and function but also establish a structural framework for future studies, including potential strategies for targeting GPIT in fungal‐specific therapeutic interventions.

## 4 Experimental Section

### Protein Expression and Purification

The codon‐optimized full‐length cDNAs of *S. cerevisia*e Gaa1 (UniProt ID: P39012‐1), Gab1 (P41733‐1), Gpi8 (P49018‐1), Gpi16 (P38875‐1) and Gpi17 (Q04080‐1) were subcloned into the pCAG vectors. A triple FLAG tag was fused to the C terminus of Gpi8. Human embryonic kidney (HEK) 293F cells (Invitrogen) were cultured in Freestyle 293 medium at 37 °C under 5% CO_2_ in a shaker at 120 r.p.m. The cultured cells at a density of ≈2 × 10^6^ cells per milliliter were co‐transfected with equal mass amounts of the plasmids. For 1‐liter cell culture, ≈2 mg of plasmids was pre‐mixed with 6 mg of PEI in 40 mL of fresh medium for 30 min at room temperature before transfection. The transfected cells were cultured at 37 °C for 48 h before harvest.

For complex purification, the transfected cells were harvested by centrifugation at 4000 g for 10 min, and lysed by sonication on ice in buffer: 25 mM Tris‐HCl pH 7.5, 150 mM NaCl and 1% protease‐inhibitor cocktail (Roche). The membrane was solubilized with 1% (w/v) n‐dodecyl‐β‐D‐maltopyranoside (DDM, Anatrace) and 0.1% cholesteryl hemisuccinate Tris salt (CHS, Anatrace) at 4 °C for 2 h. Cell debris and insoluble material were removed by ultra‐centrifugation at 20 000 g for 1 h, the collected supernatant was incubated with anti‐Flag M2 affinity resin (Sigma). The resin was rinsed with washing buffer containing 25 mM Tris‐HCl pH 7.5, 150 mM NaCl and 0.03% glyco‐diosgenin (GDN, Anatrace), and eluted with washing buffer supplemented with 200 µg mL^−1^ 3 × FLAG peptide. The protein eluate was concentrated with a 100‐kDa cut‐off Centricon (Millipore), and was further purified with SEC (Superose 6 10/300 GL, GE Healthcare) in buffer: 25 mM Tris pH 7.5, 150 mM NaCl, and 0.03% glyco‐diosgenin (GDN, Anatrace). The peak fractions were examined using SDS–PAGE with each subunit identified by mass spectrometry (MS). The fractions corresponding to monomeric or dimeric complex were pooled and concentrated to ≈5 mg mL^−1^ for cryo‐EM grid preparation. For the preparation of catalytically inactive GPIT complex, Gpi8 C199S mutation was introduced by site‐directed mutagenesis and confirmed by sequencing. The resulting inactive GPIT(Gpi8 C199S) complex was expressed and purified as the wild‐type complex described above.

### Cryo‐EM Data Acquisition

For single‐particle cryo‐EM analysis, a 3 µL aliquot of the monomeric or dimeric GPIT complex at a concentration of ≈5 mg mL^−1^ was applied to glow‐discharged Quantifoil carbon grids (R1.2/1.3 Au, 300 mesh). The grids were vitrified in liquid ethane using a FEI Vitrobot Mark IV (4 °C and 100% humidity) with a blotting time of 3 s. For the CSP‐incubated GPIT(Gpi8 C199S) sample, the purified GPIT(Gpi8 C199S) complex (≈5 mg mL^−1^) was mixed with synthesized CSP peptide (MKC7 residues 564‐596) at a 1:1.2 molar ratio. The mixture was incubated at 4 °C for one hour before freezing the cryo‐EM grids. Cryo‐EM data was collected on a FEI Titan Krios electron microscopy operated at 300 kV equipped with a Gatan K3 Summit camera and a GIF quantum energy filter. Automated data acquisition was performed with EPU (FEI) software package. Micrographs were recorded under super‐resolution counting mode with a physical pixel size of 0.92 Å or 1.06 Å. Defocus values varied from −1.1 to −3 µm. A total exposure of 1.3 s was dose‐fractionated into 32 sub‐frames, yielding a total accumulated dose of 50 electrons per Å^2^.

### Image Processing and 3D Reconstruction

The dose‐fractionated movies were subjected to motion correction and dose weighting using MotionCor2.^[^ ^46^ ^]^ CTFFIND4 was utilized to estimate the Contrast Transfer Function (CTF) parameters for each drift‐corrected micrograph.^[^ ^47^ ^]^ All subsequent image processing steps were carried out using RELION‐3.0.^[^ ^48^ ^]^ For the monomeric GPIT dataset, a set of ≈1000 particles were manually picked to generate 2D class templates for reference‐based automatic particle picking. The automatic picking yielded 464 271 particles from 3960 micrographs. Multiple rounds of 2D classification were carried out to remove low‐quality particles, resulting in a cleaned set of 207 596 particles. An ab initio map was generated using RELION and was then used as the initial reference model for 3D classification. Following two rounds of 3D classification, two 3D classes with high‐resolution features (55 376 particles) were combined for subsequent 3D refinement, particle polishing, and post‐processing. Finally, a map with an overall resolution of 3.6 Å was obtained.

For the dimeric GPIT dataset, it was processed following similar procedure as described above. Briefly, automatic particle picking yielded 535 419 particles from 4160 micrographs. One round of 2D classification resulted a cleaned set of 464 481 particles. 3D initial model was generated using RELION. After three rounds of iterative 3D classification, a 3D class (29 584 particles) with high‐resolution features for both protomers was selected for subsequent 3D refinement, particle polishing, and post‐processing. This generated a map with an overall resolution of 4.4 Å.

For the CSP‐incubated GPIT(Gpi8 C199S) dataset, automatic particle picking procedure generated a set of 977591 particles from 5495 micrographs. This particle dataset was subsequently subjected to 2D classification to remove low‐quality particles, resulting in a cleaned set of 867523 particles. The monomeric GPIT model was used as the initial reference model for further 3D classification. A 3D class with high‐resolution features (371088 particles) was selected. Subsequent particle polishing, 3D refinement and post‐processing generated a map with an overall resolution of 3.3 Å.

The overall resolution estimates were based on the gold standard Fourier shell correlation (FSC) 0.143 criterion calculations.^[^ ^49^ ^]^ The local resolution distribution was estimated using ResMap.^[^ ^50^ ^]^

### Model Building and Refinement

The atomic model of monomeric GPIT was built from the map of monomeric GPIT (with an overall resolution of 3.6 Å). The initial model generated using AlphaFold3 was rigid‐body fitted into the map using Chimera.^[^ ^51^, ^52^ ^]^ It was subsequently improved by manual rebuilding in Coot,^[^ ^53^ ^]^ guided by the good densities around the TM helices and bulky densities around residues such as Trp, Tyr, Phe and Arg. It was followed by iterative refinement in PHENIX with secondary structure and geometry restraints applied.^[^ ^54^ ^]^ For the dimeric GPIT structure, two copies of GPIT monomer were docked into the map of dimeric GPIT. Rigid‐body adjustments of each monomer, followed by rigid‐body adjustments of each subunit, were performed in Chimera to slightly optimize the fit. The final models were assessed using MOLPROBITY.^[^ ^55^ ^]^ Chimera, ChimeraX^[^ ^56^ ^]^ and PyMOL (Schrödinger, LLC.) were used to prepare the illustrated figures. Statistics of the 3D reconstructions and model refinements were provided in Table S1 (Supporting Information).

### Flow Cytometry

The *CWP2* gene was PCR‐amplified from *S. cerevisiae* genome and cloned into pRS423 plasmid. A GFP‐tag and V5 epitope were incorporated downstream of the N‐terminal signal peptide sequence of CWP2, generating the GFP‐V5‐CWP2 construct (Figure 2F). For functional analysis of the GPIT complex, modified yeast strains with chromosomal mutations in individual GPIT subunits were constructed. These strains were derived from the parental *S. cerevisiae* strain BY4742 and the mutations were introduced using the standard homologous recombination method.^[^ ^57^ ^]^

Wild‐type or GPIT mutant strains were transformed with the GFP‐V5‐CWP2 plasmid and grown overnight at 30 °C with shaking (180 r.p.m.) in SGlu‐Leu‐His (Yeast Synthetic Drop‐out Medium without Leucine and Histidine, supplemented with 2% glucose). The overnight cultures were diluted to an OD_600_ of 0.1 and incubated at 30 °C for additional 6 h. For surface labeling, cells were washed twice with PBS buffer containing 1% BSA, resuspended in 200 µL of the same buffer, and incubated with iFluor 647‐conjugated V5 tag antibody (1:250 dilution; cat. no. A01805‐100, GenScript) for 30 min on ice in the dark. After washing twice with PBS buffer, cells were resuspended in 200 µL PBS for flow cytometry analysis (Novocyte 2060R cytometer) using two wavelength pairs (488/525 nm for GFP, 645/660 nm for iFluor 647). The gating strategy was described in Figure S7A (Supporting Information). Briefly, cells (wild‐type or mutants) were initially gated to select living and single cells, followed by gating based on GFP fluorescence to identify cells expressing the GFP‐V5‐CWP2 reporter. The GFP‐positive subpopulation was subsequently analyzed for positive signals in the APC channel via iFluor 647‐conjugated V5 tag antibody, indicating surface staining of V5‐CWP2 via its V5 epitope. FACS data analysis was performed using FlowJo (BD Life Sciences). The expression level of all mutants was comparable to that of the wild‐type (WT), as evidenced by the Western blot analysis (Figure S11, Supporting Information).

### Western Blot

For western blot analysis, all five subunits of the GPIT complex were individually subcloned into pCAG vectors, with a C‐terminal FLAG tag fused into one specific subunit. HEK293F cells were cultured to a density of ≈2 × 10⁶ cells per milliliter and co‐transfected with equal mass of the plasmids using PEI. Following 48 h of incubation at 37 °C, the cells were lysed in buffer (25 mM Tris pH 7.5, 150 mM NaCl, 1% DDM, 0.1% CHS and 1% protease‐inhibitor cocktail) for 1 h at 4 °C and then centrifuged at 20 000 × g for 30 min. The samples were resuspended in SDS‐loading buffer, separated on a 15% polyacrylamide gel, and then transferred to a PVDF membrane. The membrane was subsequently immunoblotted with the indicated antibodies. Loading controls were selected based on the molecular weight of the target protein to ensure optimal band separation. Following primary mouse monoclonal antibodies were used: anti‐FLAG (Proteintech, Cat No. 66008‐4‐lg, 1:5000) for detection of the FLAG‐tagged subunit, and either anti‐β‐actin (Proteintech; Cat No. 66009‐1‐Ig,1:5000) or anti‐α‐tubulin (Proteintech; Cat No. 66031‐1‐Ig,1:5000) for loading controls. The secondary antibody was HRP‐conjugated anti‐mouse IgG (Proteintech, Cat No. SA00001‐1, 1:10000). Protein bands were visualized using enhanced chemiluminescence (ECL) with a TANON 5200CE imaging system.

### Construction of the Conditional Lethal S. Cerevisiae Strain BY4742‐HIS‐GAL‐GPI8

To circumvent the lethal phenotype caused by *GPI8* deletion,^[^ ^21^ ^]^ we constructed a conditional lethal *S. cerevisiae* strain *BY4742‐HIS‐GAL‐GPI8*. This is done by placing the expression of *GPI8* gene under the control of the stringently regulated GAL1 promoter, which is repressed in glucose‐containing medium.^[^ ^58^, ^59^ ^]^ A *HIS3*‐selectable *GAL1* promoter replacement cassette (HIS‐GAL1) was assembled and introduced into the BY4742 strain via homologous recombination, replacing the endogenous *GPI8* promoter.^[^ ^57^, ^59^ ^]^ Transformants were selected on SGal‐His (Yeast Synthetic Drop‐out Medium without Histidine, supplemented with 4% galactose). Correct integration of the HIS‐GAL1 cassette was verified by genomic PCR and DNA sequencing, yielding the conditional‐lethal strain *BY4742‐HIS‐GAL‐GPI8*.

### Spot Assay for Growth Phenotypic Analysis of GPI8 Variants

To assess the functional consequences of *GPI8* variants, spot growth assays using the conditional‐lethal strain *BY4742‐HIS‐GAL‐GPI8* constructed above was performed. This strain was transformed with the empty vector pRS424, pRS424 with wild‐type *GPI8*, or pRS424 with *GPI8* variants. Transformants were selected on SGal‐Leu (Yeast Synthetic Drop‐out Medium without Leucine, supplemented with 4% galactose) and verified by PCR and DNA sequencing. The confirmed transformants were cultured in SGal‐Leu at 30 °C with shaking (180 r.p.m.) until reaching exponential phase (OD_600_ = 1.0). Cells were serially diluted 10‐fold, and 5 µL of each dilution was spotted on SGal‐Leu or SGlu‐Leu (Yeast Synthetic Drop‐out Medium without Leucine, supplemented with 2% glucose). After incubation at 30 °C for 72 h, plates were photographed with the 5200CE Image System (TANON). Each assay was repeated three times with similar results.

### Protein Identification by Mass Spectrometry

Protein samples were separated by SDS–polyacrylamide gel electrophoresis and visualized by Coomassie staining. Protein bands of interest were manually excised and destained, followed by reduction with dithiothreitol (DTT) and alkylation with iodoacetamide (IAA). Proteins were then digested overnight at 37 °C with proteomics‐grade trypsin in 20 mM ammonium bicarbonate. Digestion was quenched by the addition of 2% formic acid. Peptides in gel were extracted using solution containing 2% formic acid and 67% acetonitrile, vacuum‐dried and resuspended in 0.1% formic acid. Peptide mixtures were loaded onto a nanoViper C18 trap column (3 µm, 100 Å) and separated on an analytical column (Acclaim PepMap RSLC, 75 µm × 25 cm; C18, 2 µm, 100 Å) using an EASY‐nLC 1200 system (Thermo Fisher Scientific). Peptides were eluted with a linear gradient of 5%–38% buffer A (80% acetonitrile and 0.1% formic acid) over 30 min. Mass spectrometry analysis was conducted on a Q Exactive mass spectrometer (Thermo Fisher). The acquired data were converted to mgf format using ProteoWizard and subsequently analyzed with the Mascot search engine against the UniProt Saccharomyces cerevisiae database with a false discovery rate of less than 1%.

## Conflict of Interest

The authors declare no conflict of interest.

## Author Contributions

M.Z. and H.Y. conceived the project. M.Z., M.J.Z., X.L. and H.Y. designed experiments. Z.H., X.D., X.L. performed most of the experiments. Y.W., D.Z., X.H., P.Y., J.L., Y.T. and J.L. helped with the sample preparation, functional experiments and data collection. Z.H., X.D., M.J.Z., M.Z., X.L. and H.Y. analyzed the data. M.Z. and H.Y. wrote the manuscript with the help of all the authors. Z.H., X.D. contributed equally to this work.

## Supporting information



Supporting Information

## Data Availability

The data that support the findings of this study are available in the supplementary material of this article.
