# Supplementary material for: Molecular Insights into Fungal Glycosylphosphatidylinositol Transamidase Complex
Source: Adv Sci (Weinh). 2025 Oct 14;13(2):e11340. doi: 10.1002/advs.202511340 (PMC12786357; doi:10.1002/advs.202511340)
Supplement: Supplementary file 1 — Supporting Information [file ADVS-13-e11340-s001.docx]

Supporting Information

**Molecular insights into fungal glycosylphosphatidylinositol transamidase complex**

*Zhengkang Hua, Xuyang Ding, Yanan Wu, Di Zhang, Xinlin Hu, Ping Yang, Jiameng Li, Yi Tan, Junbo Liu, Mingjie Zhang, Min Zhang***, Xiaotian Liu***, Hongjun Yu**

**
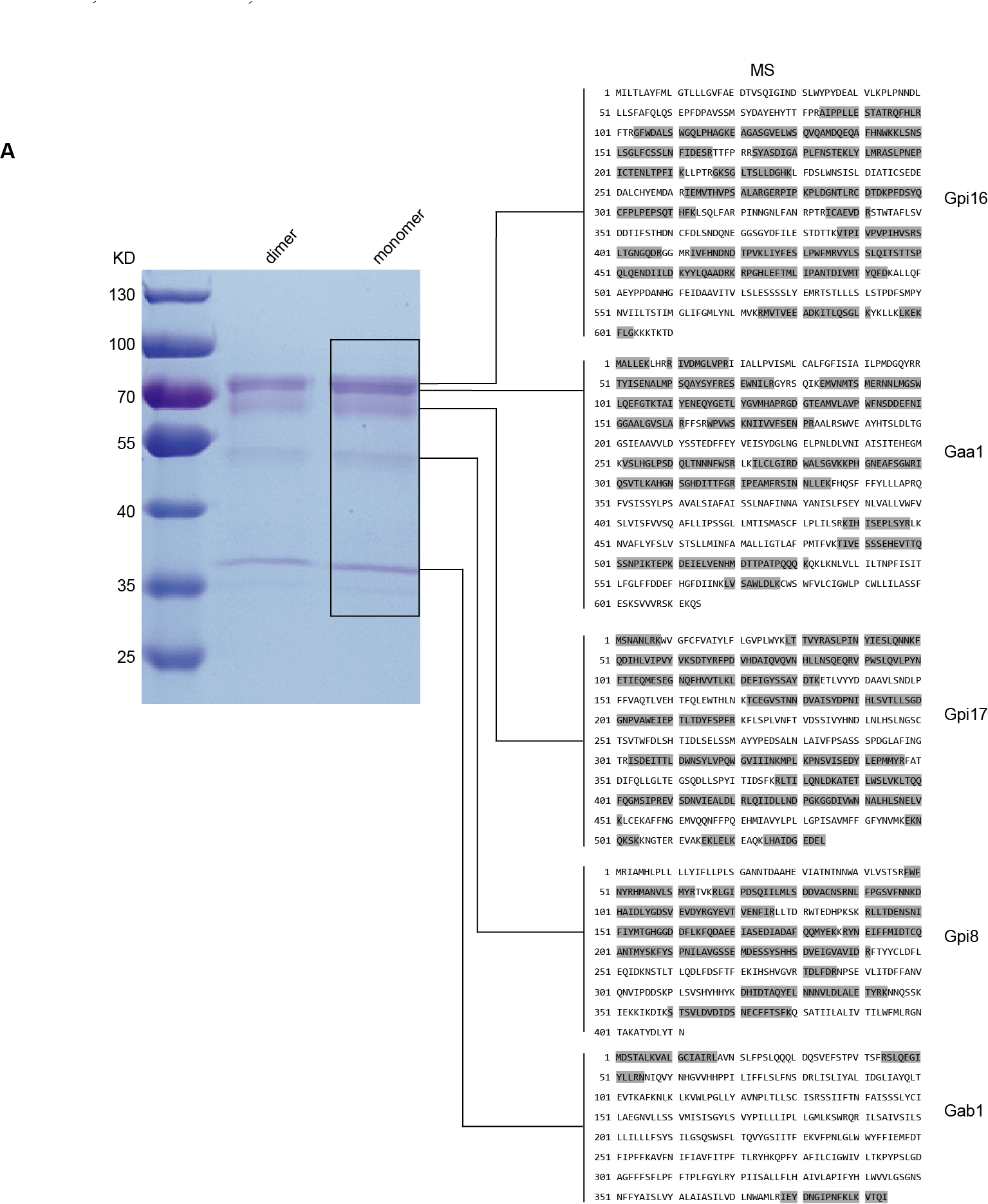
**

**Figure S1. Mass spectrometric identification of GPIT subunits.**

**A,** SDS-PAGE analysis of purified monomeric and dimeric GPIT complex, followed by mass spectrometry analysis of corresponding bands. All five subunits were identified with detected peptides for each subunit highlighted in grey.

**
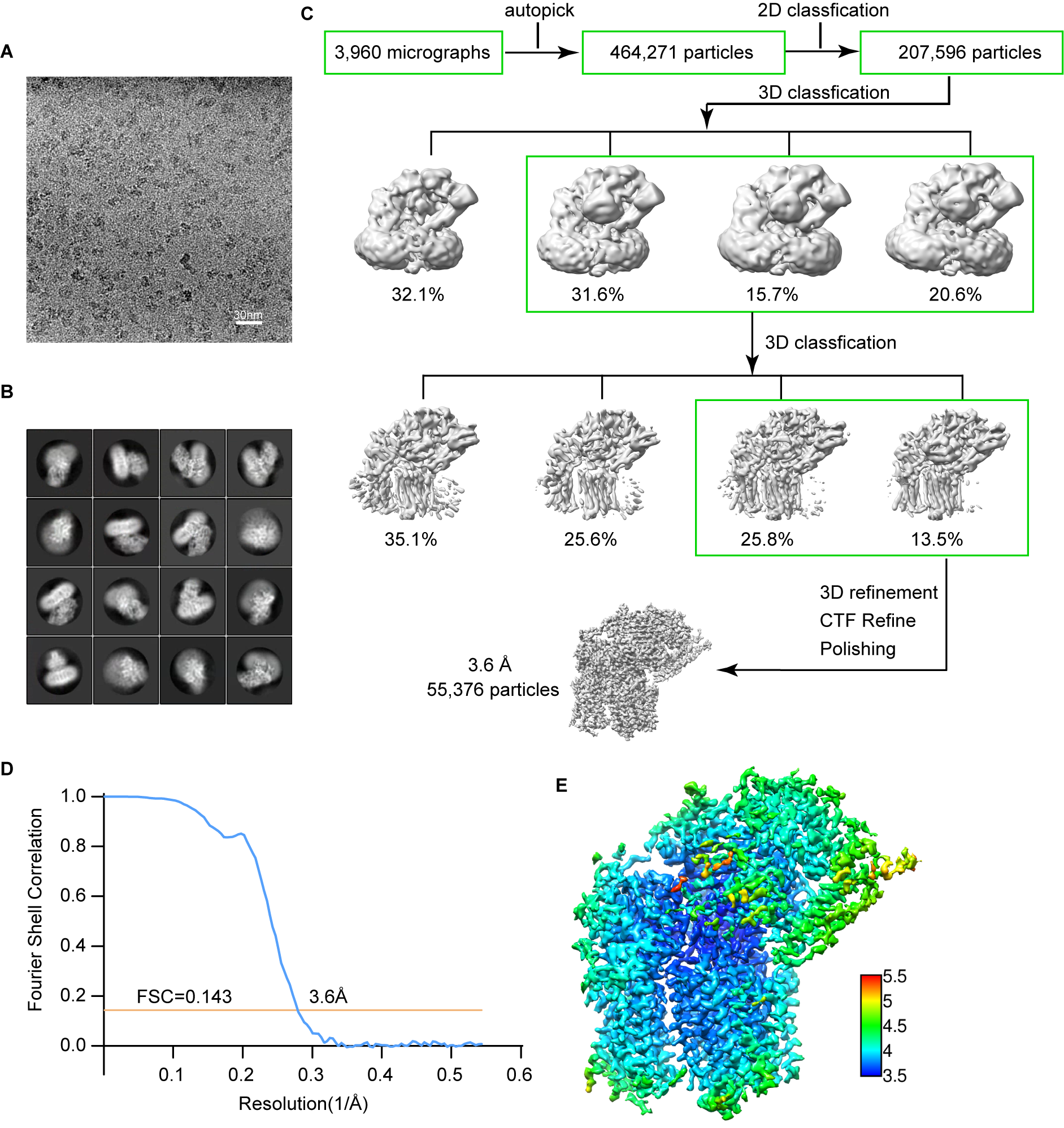
**

**Figure S2. Cryo-EM analysis of monomeric GPIT.**

**A,** A representative cryo-EM micrograph of monomeric GPIT.

**B,** Representative 2D class averages.

**C,** Flowchart for cryo-EM data acquisition and data processing of monomeric GPIT. See Methods for more details.

**D,** The gold-standard Fourier shell correlation (FSC) curve of the reconstructed map.

**E,** Local resolution distribution of the final cryo-EM map of monomeric GPIT.


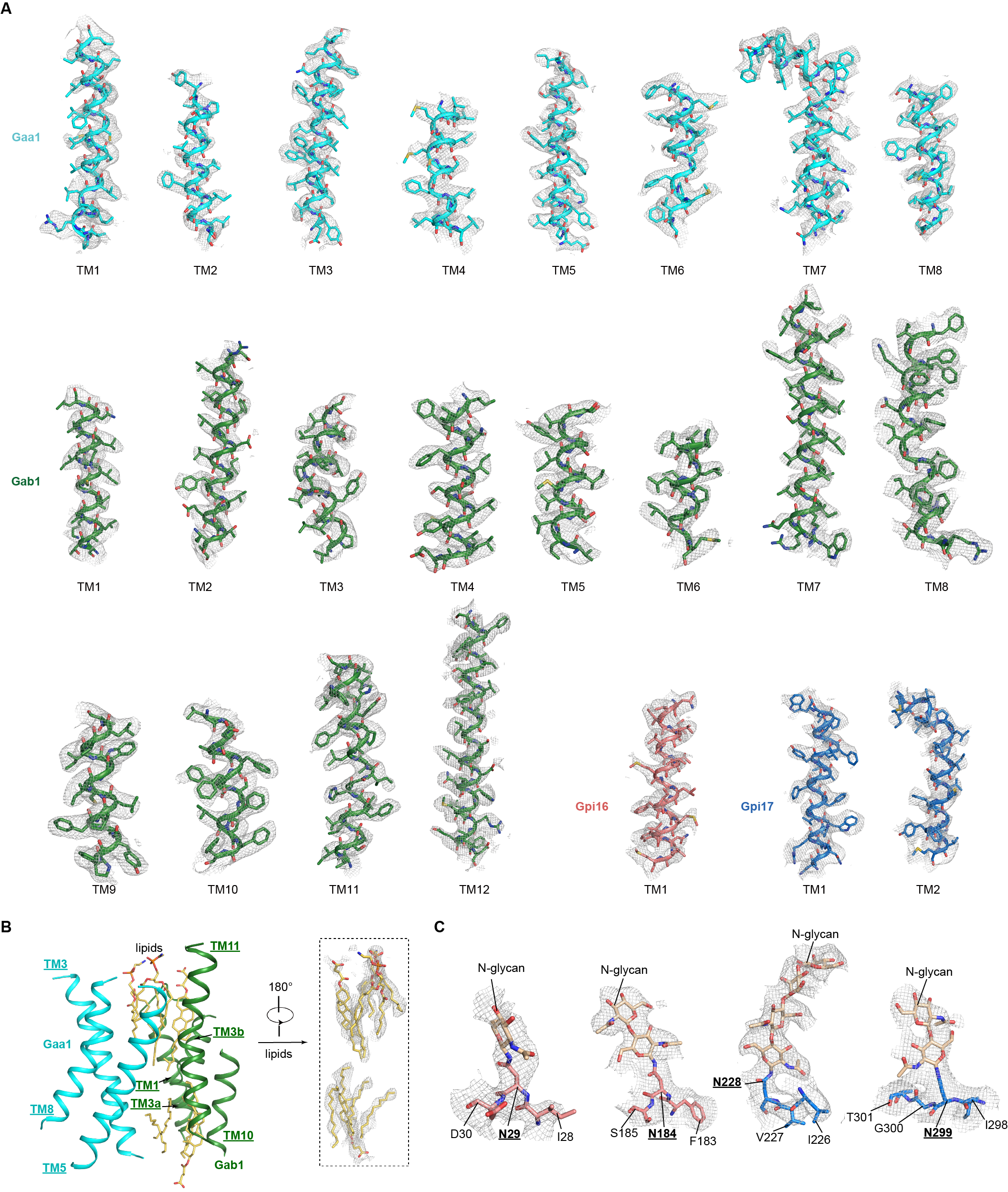


**Figure S3. Fit of cryo-EM map with the monomeric GPIT model in example regions.**

**A,** Cryo-EM densities of the transmembrane (TM) helices for all subunits of monomeric GPIT.

**B,** Cryo-EM densities of the ordered lipids at the transmembrane interface between Gaa1 and Gab1.

**C,** Cryo-EM densities of the N-glycans identified with GPIT.

**
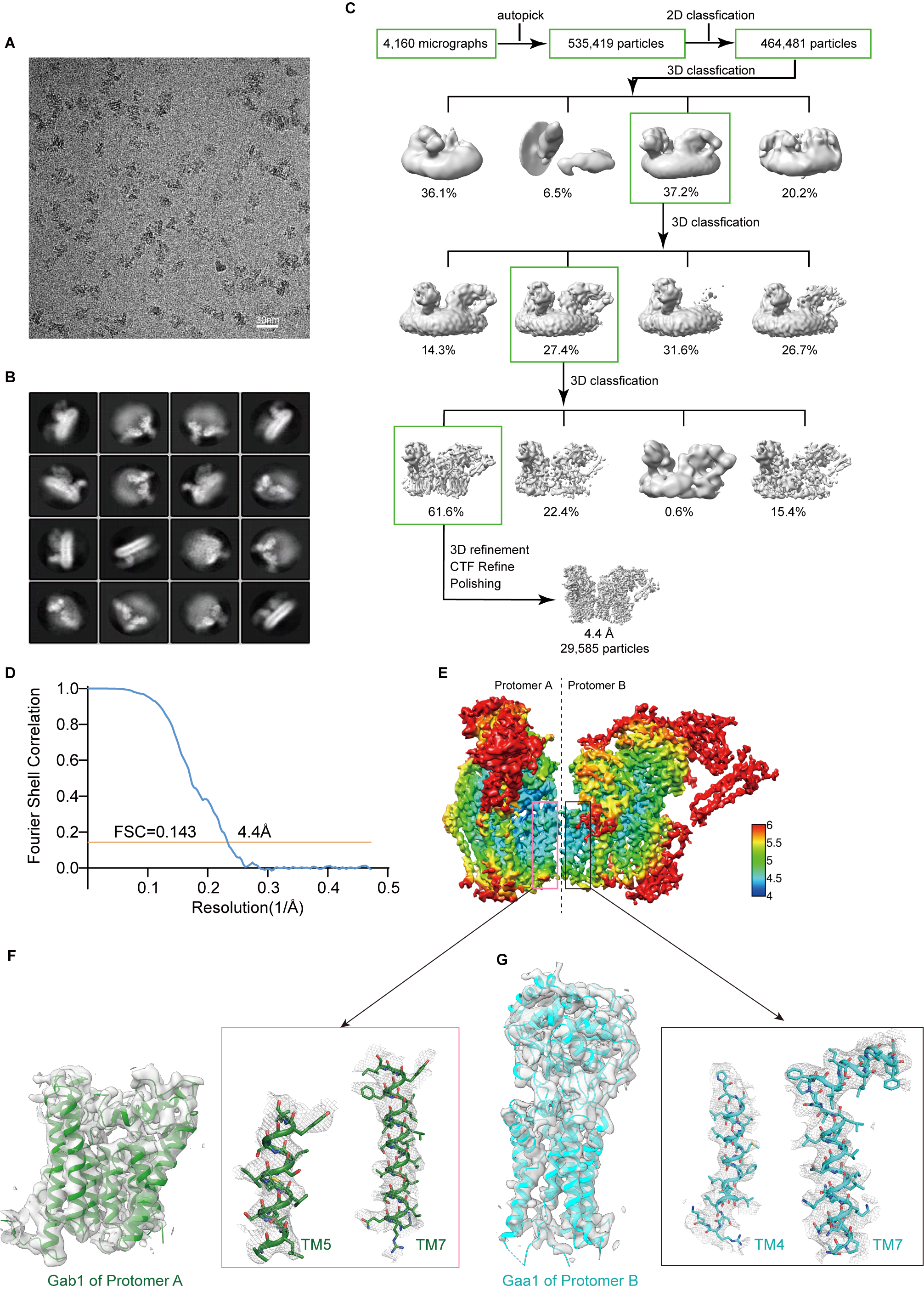
**

**Figure S4. Cryo-EM analysis of dimeric GPIT.**

**A,** A representative cryo-EM micrograph of dimeric GPIT.

**B,** Representative 2D class averages.

**C,** Flowchart for cryo-EM data acquisition and data processing of dimeric GPIT. See Methods for more details.

**D,** The gold-standard Fourier shell correlation (FSC) curve of the reconstructed map.

**E,** Local resolution distribution of the final cryo-EM map of dimeric GPIT.

**F-G,** Fit of cryo-EM map with the dimeric GPIT model in example regions at the dimeric interface (see Figure 5A-C for more details about dimer). **F,** Cryo-EM densities of Gab1 from protomer A of dimeric GPIT. Inset (pink box), the densities corresponding to Gab1 TM5 and TM7 at the dimer interface. **G**, Cryo-EM densities of Gaa1 from protomer B of dimeric GPIT. Inset (black box), the densities corresponding to Gaa1 TM4 and TM7 at the dimer interface.

**
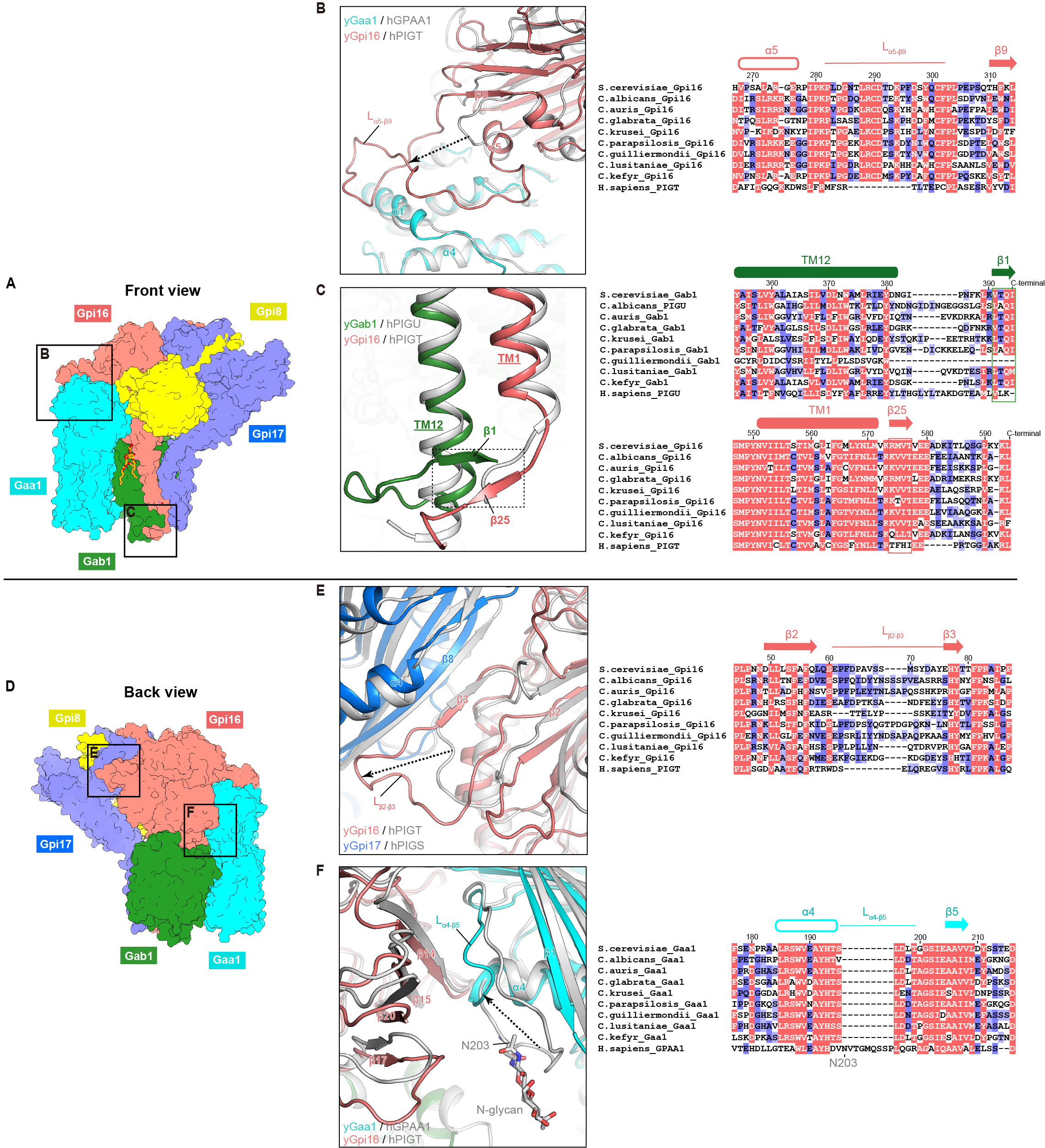
**

**Figure S5. Structure and sequence comparison of the subunit interfaces between fungal and human GPIT.**

**A,** Front view of yGPIT complex.

**B-C,** Structural comparisons (left rank) and sequence alignments (right rank) of yGPIT subunits with their corresponding hGPIT subunits, focusing on the positions indicated in (**A**). Key structural differences are marked by arrow (**B**) or dashed box (**C**). The analyzed structural elements are labelled in the sequence alignment.

**D,** Back view of yGPIT complex.

**E-F,** Structural comparisons (left rank) and sequence alignments (right rank) of yGPIT subunits with their corresponding hGPIT subunits, focusing on positions indicated in (**D**). Key structural differences are marked by arrows (**E** and **F**). The analyzed structural elements are labelled in the sequence alignment.

For the sequence alignment in (**B-C**, **E-F**), analyses were performed across human and fungal species, including *S. cerevisiae* and fungal pathogens, such as *C. albicans*, *C. auris*, *C. glabrata*, *C. krusei*, *C. parapsilosis*, *C. guilliermondii*, *C. lusitaniae*, *C. kefyr*.


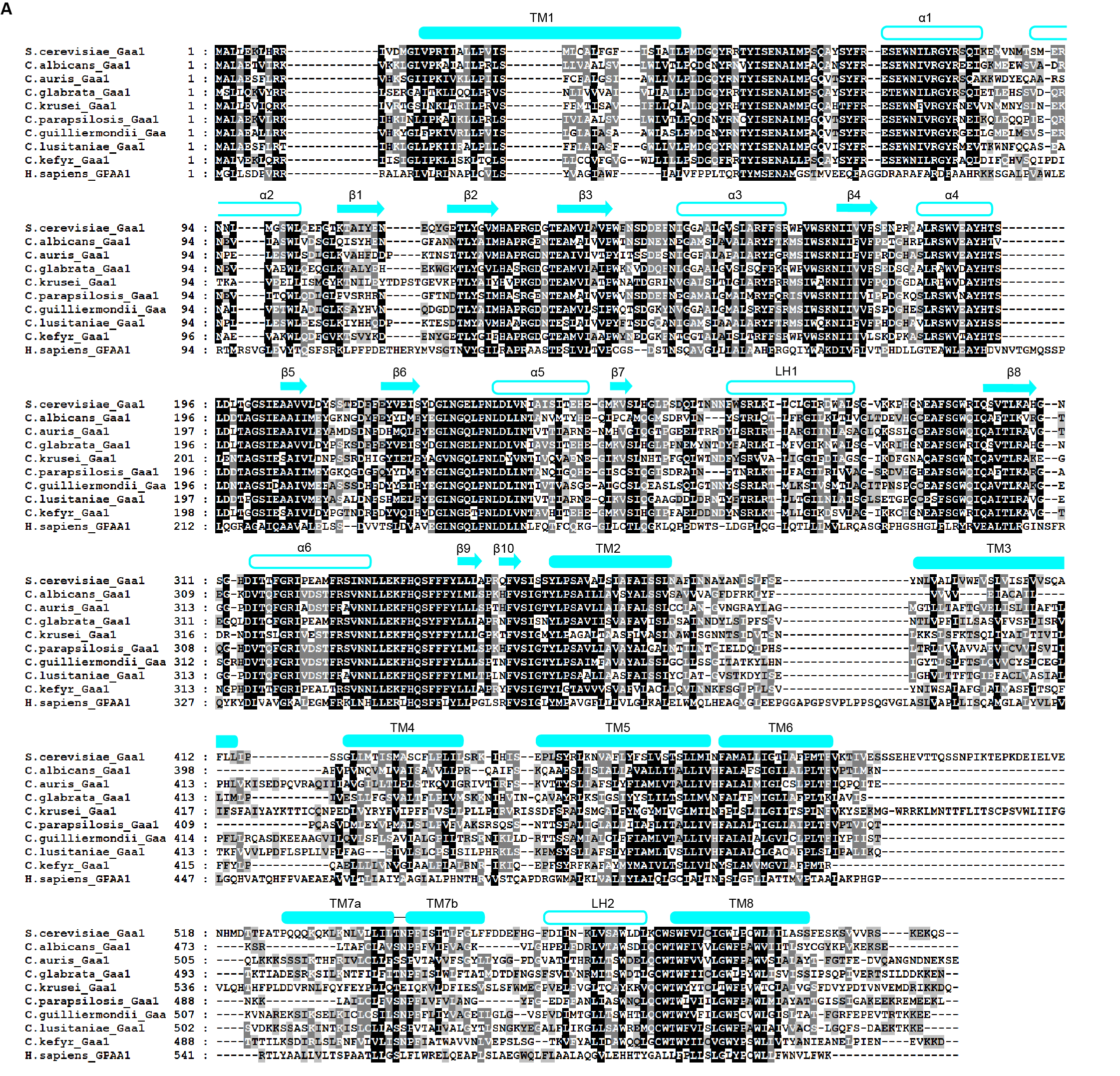

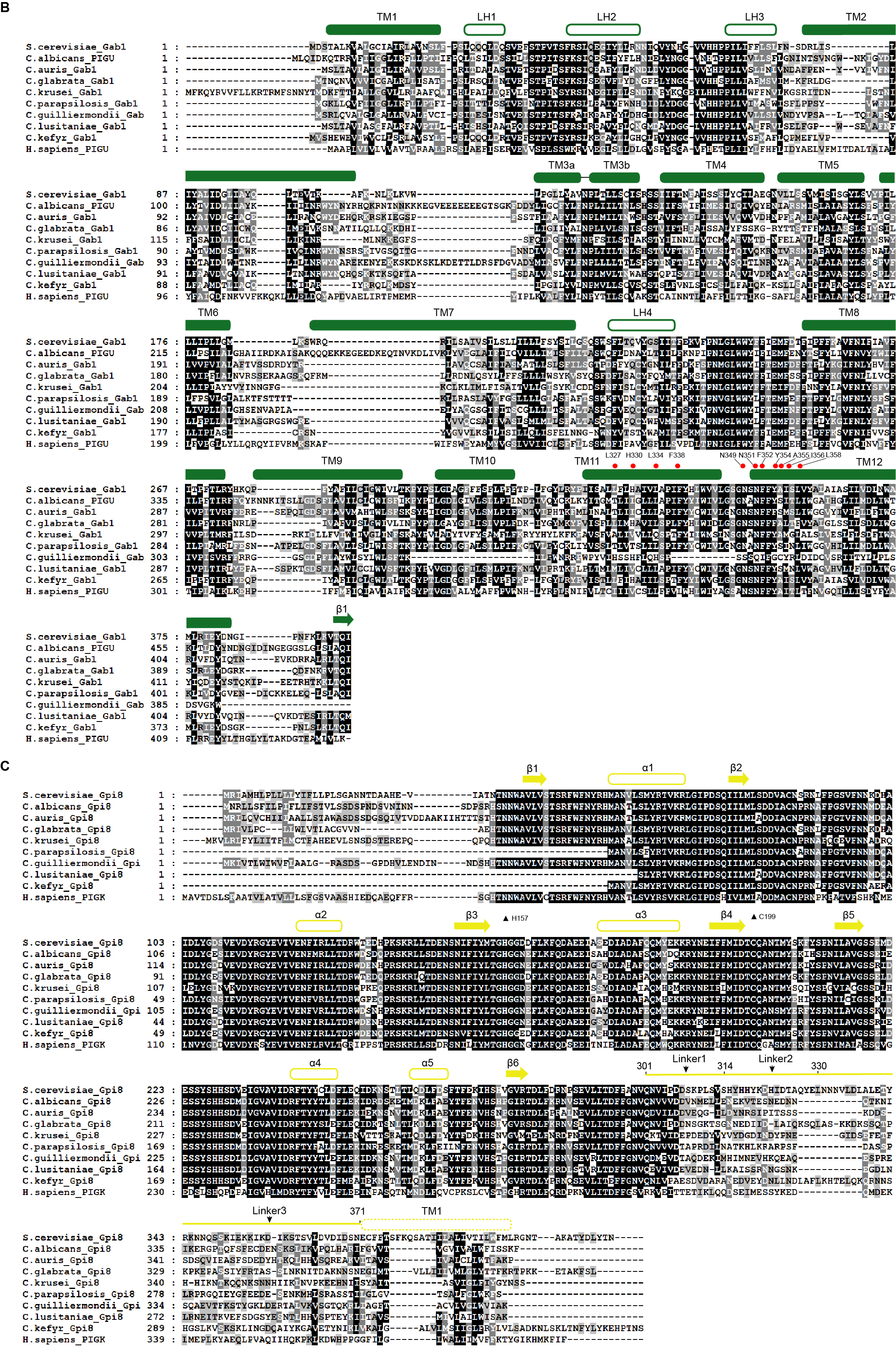

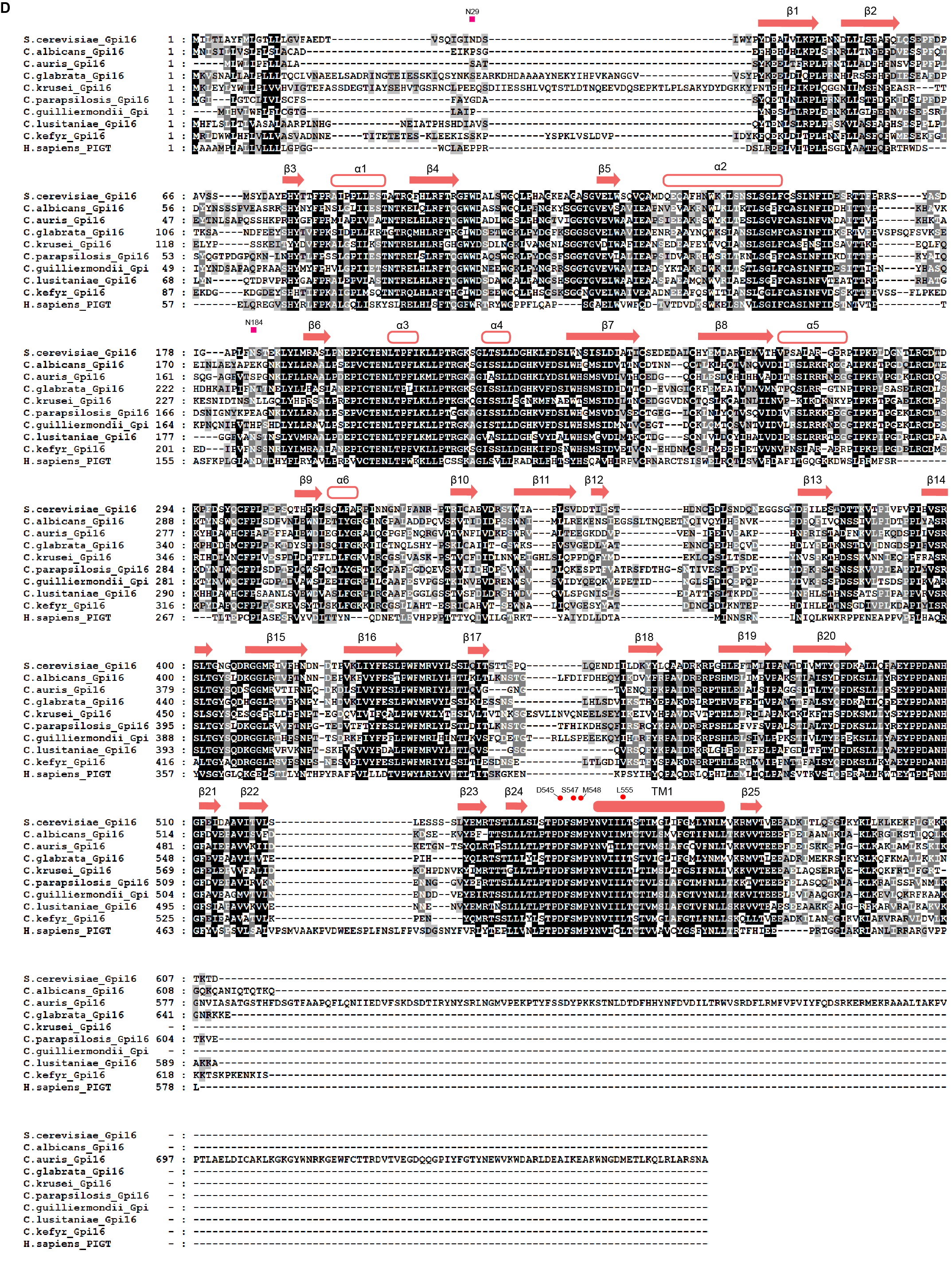

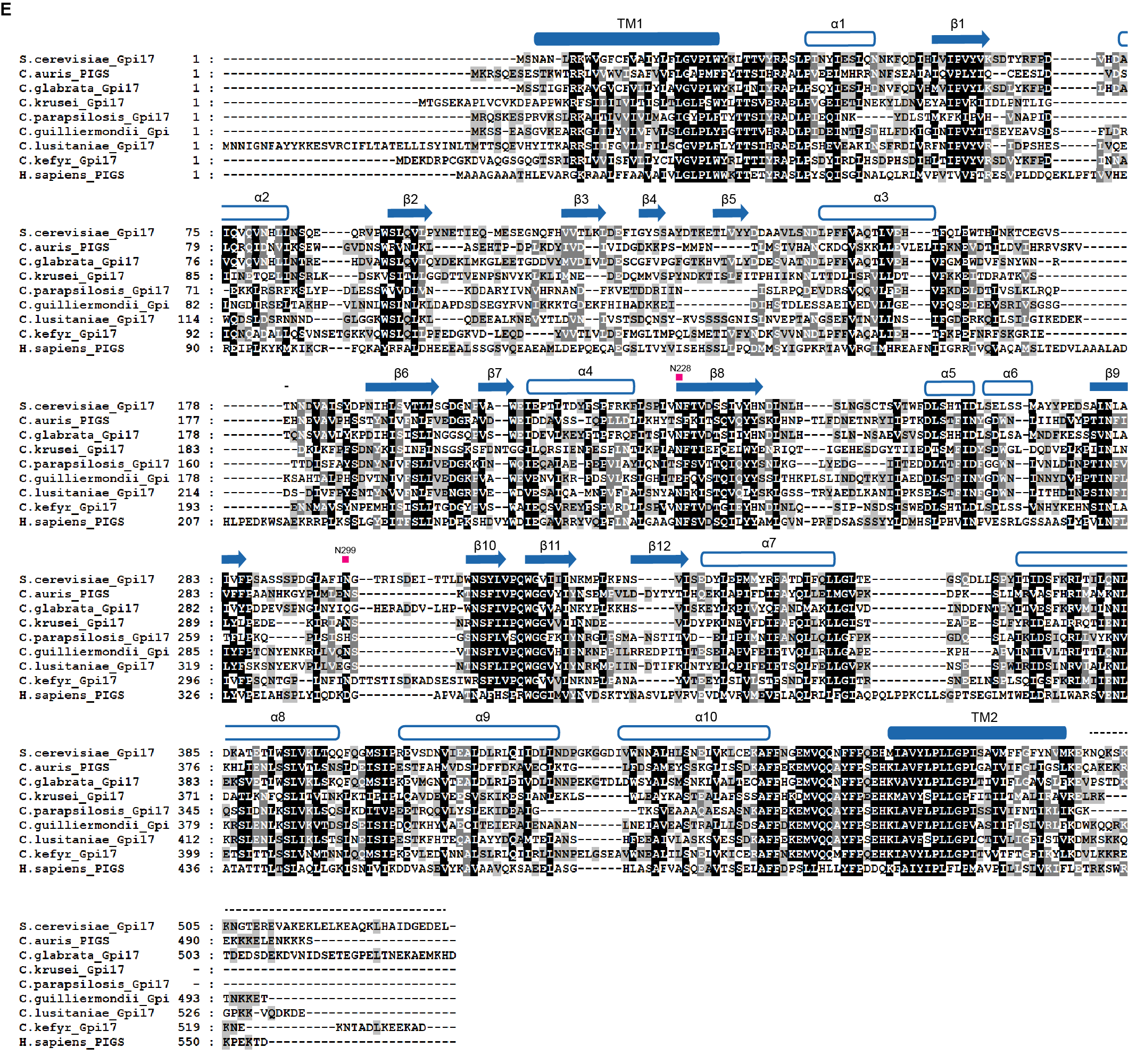


**Figure S6. Multiple sequence alignments of GPIT subunits across human and fungal species.**

The primary sequence alignment was generated using Clustal Omega and visualized using GeneDoc. Functional residues are selectively labelled, including: black triangles marking the catalytic dyad in Gpi8; red circles marking the GPI binding site; pink squares marking the glycosylation sites. Secondary structural elements, derived from the yGPIT structure, are shown above the alignments. Protein sequences are sourced from *Homo sapiens* and multiple fungal species, including *S. cerevisiae* and fungal pathogens (*C. albicans*, *C. auris*, *C. glabrata*, *C. krusei*, *C. parapsilosis*, *C. guilliermondii*, *C. lusitaniae*, *C. kefyr*).

**
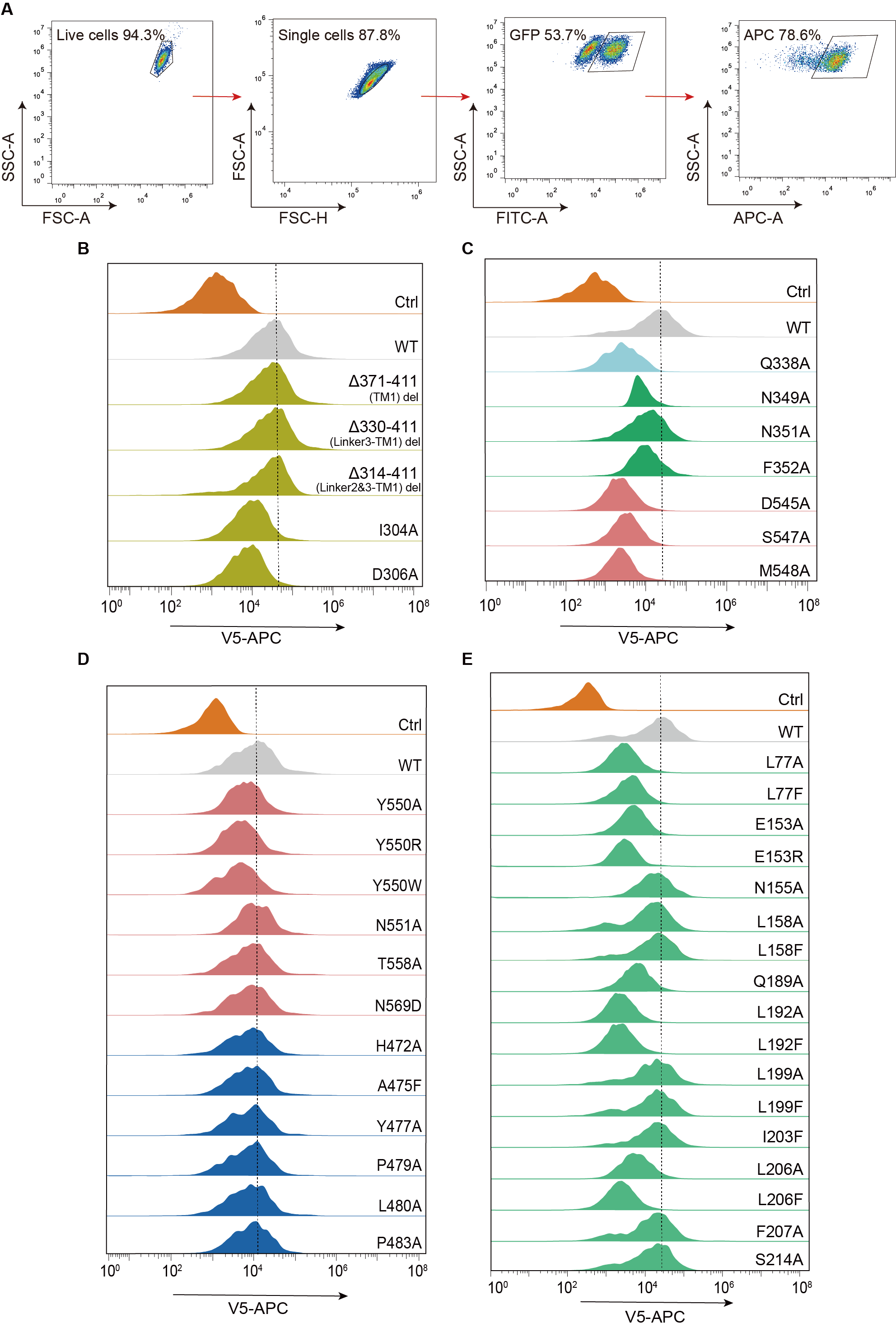
**

**Figure S7. Representative FACS (fluorescence-activated cell sorting) results for the in vivo functional analysis of GPIT mutations.**

**A,** Representative flow cytometry gating strategy. Strains carrying indicated chromosomal mutations of GPIT subunits were transformed with a plasmid expressing the GFP-V5-CWP2 construct (Figure 2F). Residual GPIT activity was quantified via FACS-based surface expression analysis of CWP2 (see methods). Cells (wild-type or mutants) were initially gated to select living and single cells, followed by gating based on GFP fluorescence to identify cells expressing the GFP-V5-CWP2 reporter. The GFP-positive subpopulation was subsequently analyzed for positive signals in the APC channel via iFluor 647-conjugated V5 tag antibody, indicating surface staining of V5-CWP2 via its V5 epitope. FSC-A/H: Forward scatter area/height; SSC-A: Side scatter area; FITC: Channel for GFP detection; APC: Channel for iFluor 647-conjugated V5 tag antibody detection.

**B-E,** In vivo functional analysis of GPIT mutations relative to the wild-type and control, with representative FACS results from three independent replicates shown. The control (orange) represents the V5-staining (iFluor 647) background of yeast cells without the transformation of GFP-V5-CWP2 construct. **B,** C-terminal truncations and mutations of Gpi8. **C,** Mutations of GPI binding site. **D,** Mutations of CSP binding site. **E,** Dimer interface mutations.

**
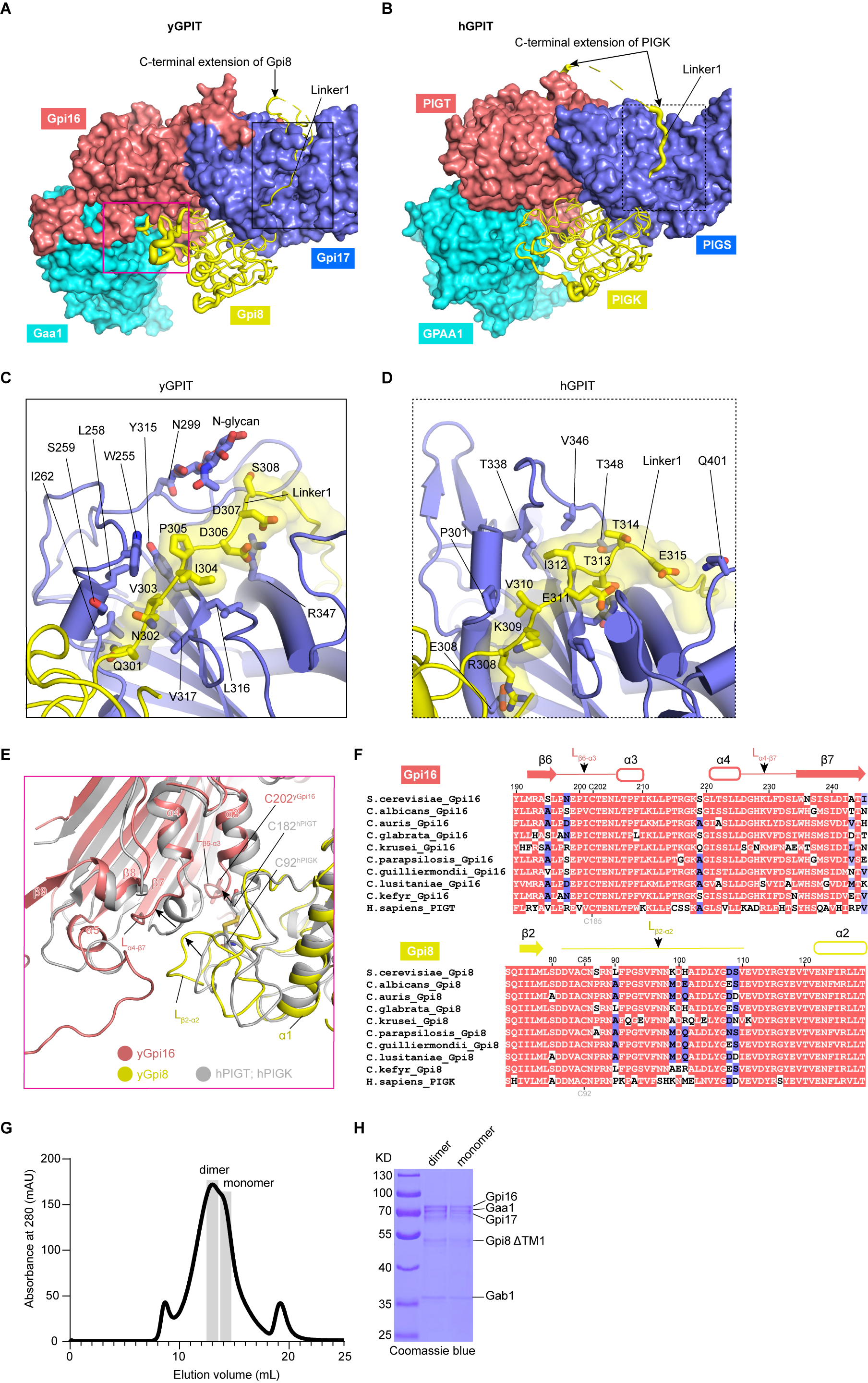
**

**Figure S8. Comparison of the binding of catalytic subunits (Gpi8 and its human homolog PIGK) between fungal and human GPIT.**

**A-B,** Different binding modes of yGpi8 and hPIGK in fungal and human GPIT. yGpi8 features a dynamic interface with yGpi16 (outlined by the pink box), characterized by high B-factors. Moreover, the C-terminal extensions of yGpi8 and hPIGK are anchored differently, as indicated by arrows. Both yGpi8 and hPIGK are represented in B-factor putty mode in PyMOL, with protein backbone thickness proportional to B-factor values. The remaining subunits are displayed in surface representations as labelled.

**C-D,** Zoomed-in, side-by-side comparison of the Gpi8 Linker1 binding site in yGPIT (**C**) and the corresponding hPIGK Linker1 binding site in hGPIT (**D**), corresponding to the boxed areas in (**A**) and (**B**), respectively.

**E-F,** Structural comparisons (**E**) and sequence alignments (**F**) of yGPIT subunits with their corresponding hGPIT subunits, focusing on the dynamic interacting interface of yGpi8 (highlighted as pink box in **A**). Key structural differences are marked by arrows in (**E**), with corresponding sequences labelled in the sequence alignment (**F**). The interfacial loops are more conserved in fungal species than in humans. Notably, though both individual cysteine residues are conserved, the inter-subunit disulfide bond between hPIGK C92 and hPIGT C182 is not formed in yGPIT, due to the retracted helix containing yGpi16 C202.

**G-H,** Elution profile (**G**) and SDS-PAGE analysis (**H**) of the SEC (Superose 6 10/300 GL)-purified GPIT complex with the Gpi8 TM1 deleted. Grey boxes mark the fractions corresponding to the monomeric and dimeric states, respectively.


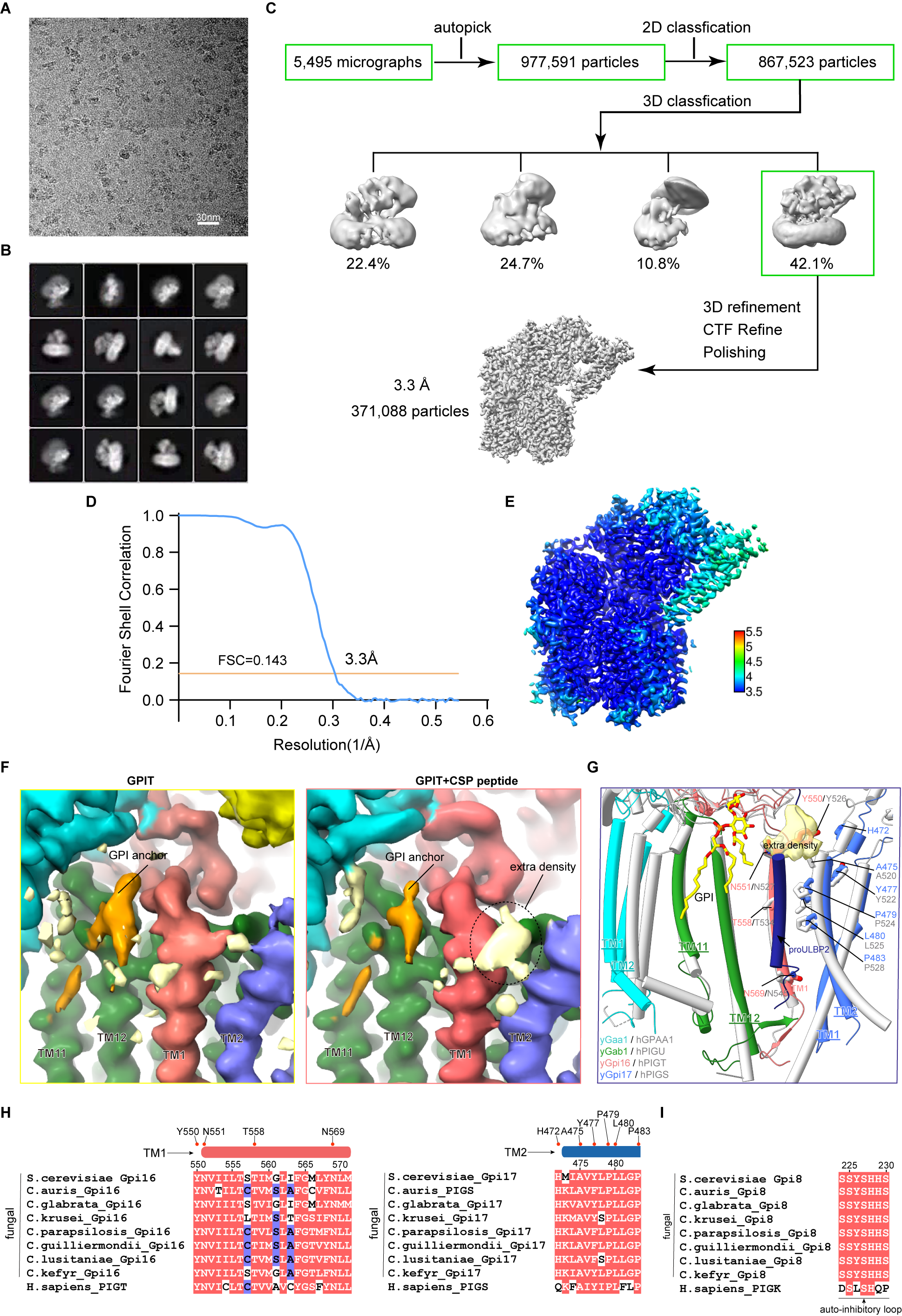


**Figure S9. Cryo-EM analysis of the complex of GPIT and CSP peptide.**

**A,** A representative cryo-EM micrograph of the complex of GPIT and CSP peptide (derived from GPI-anchored protein MKC7). See Methods and Figure 4E for details on the preparation process and purification profile of the complex.

**B,** Representative 2D class averages of the complex.

**C,** Flowchart for cryo-EM data acquisition and data processing of the complex of GPIT and CSP peptide. See Methods for more details.

**D,** The gold-standard Fourier shell correlation (FSC) curve of the reconstructed map.

**E,** Local resolution distribution of the final cryo-EM map.

**F,** Comparison of cryo-EM densities between the 3D reconstructions of GPIT (left) and GPIT with CSP peptide incubation (right). An extra density outlined by the dashed circle is observed in the latter, likely corresponding to the bound CSP peptide.

**G,** Overlay of the protein substrate binding sites between yGPIT (colored as labelled) and hGPIT/proULBP2 complex (PDB ID: 8IMY; hGPIT subunits colored in grey; proULBP2 colored in dark blue). The extra density in the reconstruction of yGPIT/CSP peptide, as revealed in (**F**), coincides with part of the proULBP2 ligand in hGPIT/proULBP2 complex, suggesting a key binding site for protein substrates conserved across human and fungal species.

**H-I,** Multiple sequence alignment of putative CSP binding sites (**H**) and autoinhibitory loop (**I**) across human and fungal species, including *S. cerevisiae* and fungal pathogens (*C. auris*, *C. glabrata*, *C. krusei*, *C. parapsilosis*, *C. guilliermondii*, *C. lusitaniae*, *C. kefyr*).


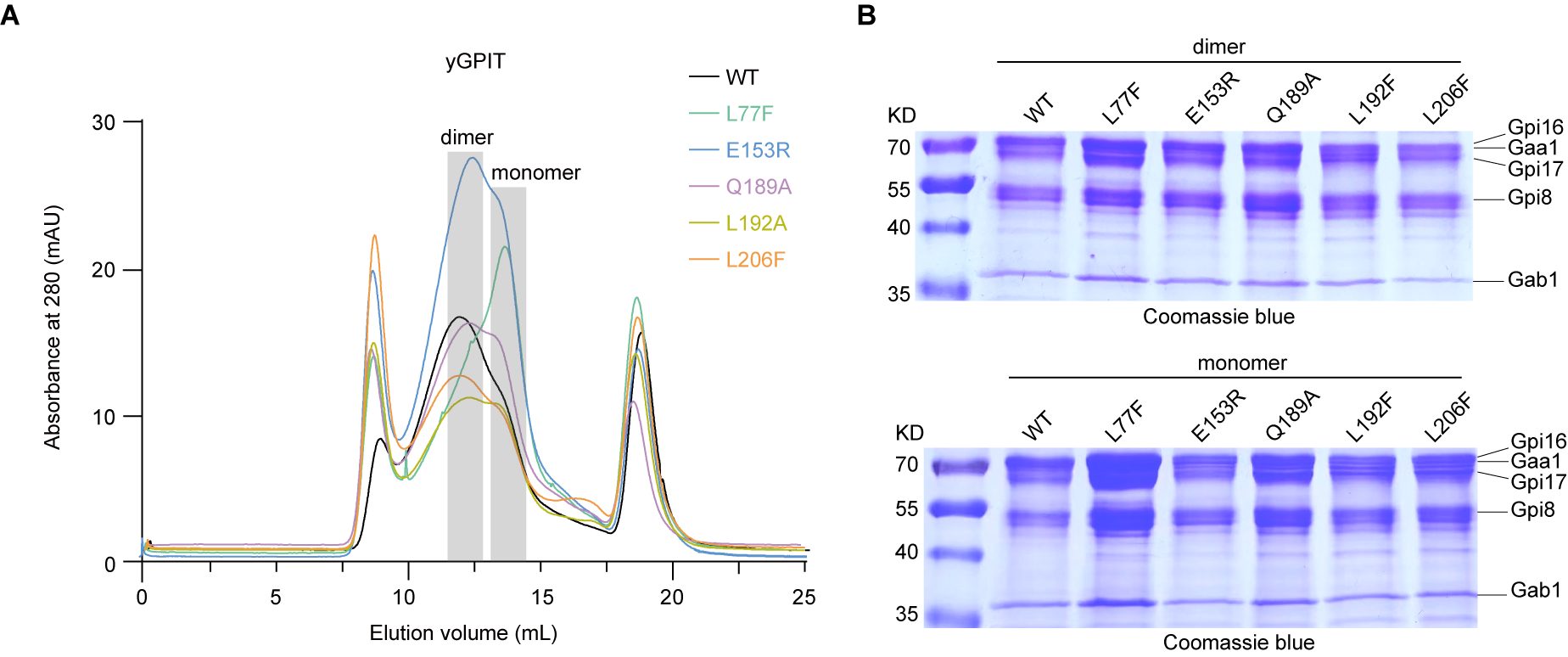


**Figure S10. Evaluation of oligomeric states of yGPIT dimeric interface mutants.**

**A,** SEC (Superose 6 10/300 GL) elution profile of purified wild-type (WT) yGPIT and yGPIT variants with indicated dimer interface mutants (L77F, E153R, Q189A, L192F, L206F), which were identified as functionally impactful in Figure 5D.

**B,** SDS–PAGE of peak fractions corresponding to dimeric (upper) and monomeric (lower) forms of yGPIT variants analyzed in (**A**).


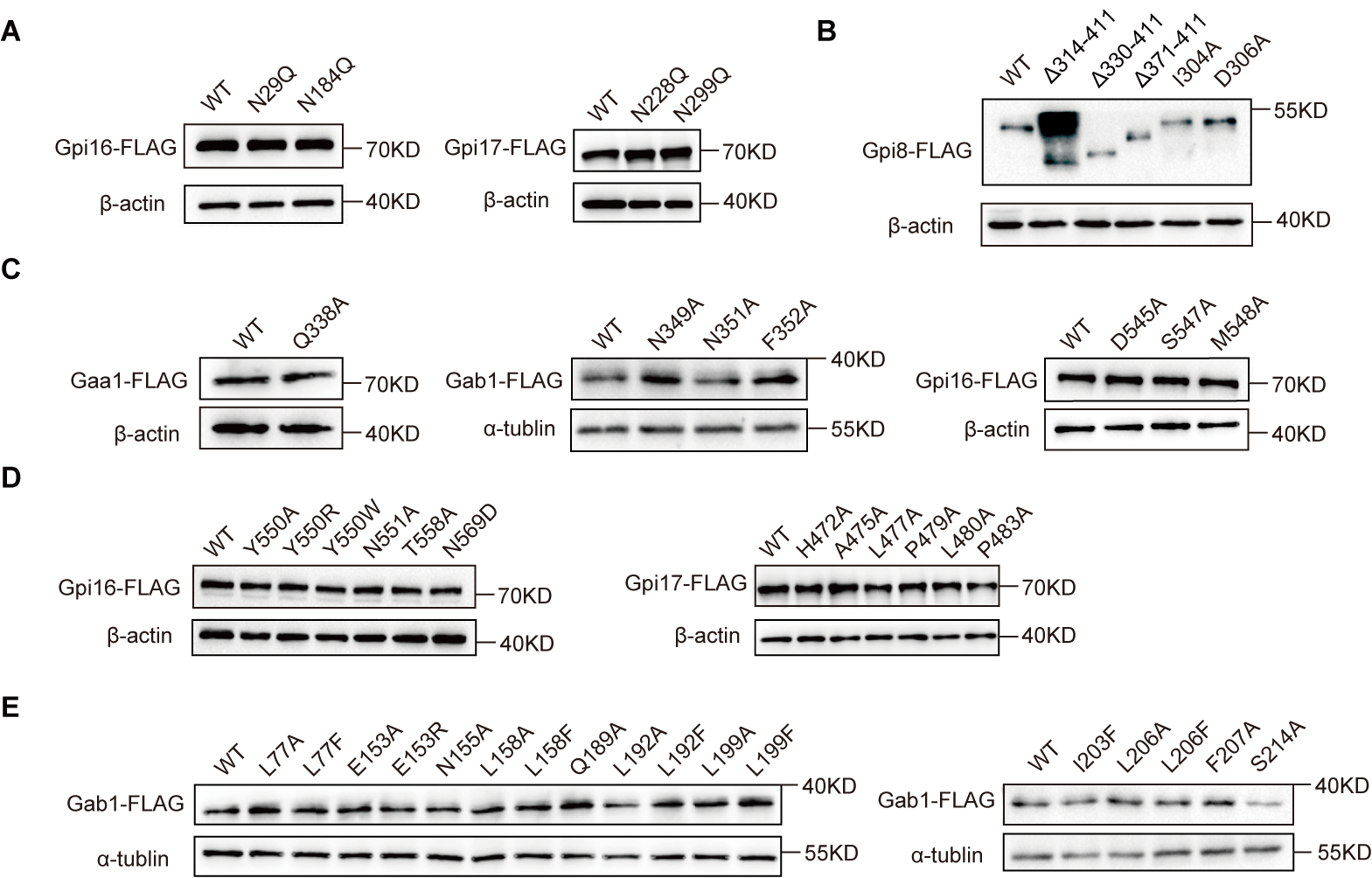


**Figure S11.** **Western blot analysis of the expression levels of GPIT and its mutants.** For loading controls, β-actin or α-tubulin was selected according to the molecular weight of the target protein to ensure optimal band separation.

**A,** N-glycosylation site mutants (as analyzed in Figure 2G).

**B,** C-terminal truncations and mutations of Gpi8 (as analyzed in Figure 3G).

**C,** Mutations of GPI binding site (as analyzed in Figure 4D).

**D,** Mutations of CSP binding site (as analyzed in Figure 4G).

**E,** Dimer interface mutations (as analyzed in Figure 5D).

**Table S1. Cryo-EM data collection, refinement and validation statistics.**

|  | **Monomeric GPIT**  **sharpened**  **map**  **(EMDB-64000)**  **(PDB 9UB7)** | | **Monomeric GPIT**  **unsharpened map**  **(EMDB-64001)** | | | **Dimeric**  **GPIT**  **sharpened**  **map**  **(EMDB-64002)**  **(PDB 9UB8)** | | | **GPIT-CSP complex**  **unsharpened map**  **(EMDB-64003)** | | |
| --- | --- | --- | --- | --- | --- | --- | --- | --- | --- | --- | --- |
| **Data collection and processing**  **Processing** | |  | |  | | |  | | |  | |
| Magnification | | 130,000 | | | 130,000 | | | 130,000 | | | 130,000 |
| Voltage (kV) | | 300 | | | 300 | | | 300 | | | 300 |
| Electron exposure (e−/Å²) | | 50 | | | 50 | | | 50 | | | 50 |
| Defocus range (μm) | | -1.1 to -3 | | | -1.1 to -3 | | | -1.1 to -3 | | | -1.1 to -3 |
| Pixel size (Å) | | 0.92 | | | 0.92 | | | 1.06 | | | 0.92 |
| Symmetry imposed | | C1 | | | C1 | | | C1 | | | C1 |
| Initial particle images (no.) | | 464,271 | | | 464,271 | | | 535,419 | | | 867,523 |
| Final particle images (no.) | | 55,376 | | | 55,376 | | | 29,585 | | | 371,008 |
| Map resolution (Å) | | 3.6 | | |  | | | 4.4 | | |  |
| FSC threshold | | 0.143 | | |  | | | 0.143 | | |  |
| Map resolution range (Å)* | | 3.5-5.5 | | |  | | | 4-6 | | |  |
|  | |  | | |  | | |  | | |  |
| **Refinement** | |  | | |  | | |  | | |  |
| Initial model used (PDB code) | | AlphaFold3 | | |  | | | This study | | |  |
| Model resolution (Å) | | 3.7 | | |  | | | 4.6 | | |  |
| FSC threshold | | 0.5 | | |  | | | 0.5 | | |  |
| Map sharpening B factor (Å²) | | -109.38 | | |  | | | -125.99 | | |  |
| Model composition | |  | | |  | | |  | | |  |
| Non-hydrogen atoms | | 18348 | | |  | | | 31758 | | |  |
| Protein residues | | 2272 | | |  | | | 3976 | | |  |
| Ligands | | 11 Lipids;  4 N-glycans;  1 GPI | | |  | | | n/a | | |  |
| *B* factor (Å^2^) | |  | | |  | | |  | | |  |
| Protein | | 63.79 | | |  | | | 138.97 | |  | |
| Ligand | | 64.78 | | |  | | | n/a | |  | |
| R.m.s. deviations | |  | | |  | | |  | |  | |
| Bond lengths (Å) | | 0.004 | | |  | | | 0.005 | |  | |
| Bond angles (°) | | 0.996 | | |  | | | 1.051 | |  | |
| Validation | |  | | |  | | |  | |  | |
| MolProbity score | | 1.64 | | |  | | | 1.97 | |  | |
| Clashscore | | 5.40 | | |  | | | 12.65 |  | | |
| Poor rotamers (%) | | 0.15 | | |  | | | 0.06 |  | | |
| Ramachandran plot | |  | | |  | | |  |  | | |
| Favored (%) | | 95.03 | | |  | | | 94.83 |  | | |
| Allowed (%) | | 4.93 | | |  | | | 5.15 |  | | |
| Outliers (%) | | 0.04 | | |  | | | 0.00 |  | | |
|  | |  | | |  | |  | |  | | |

**Table S2. Sequence conservation of GPIT subunits between fungi and mammal.**

| **Organism** | **Gaa1** | **Gab1** | **Gpi8** | **Gpi16** | **Gpi17** | **Average** |
| --- | --- | --- | --- | --- | --- | --- |
| *Saccharomyces cerevisiae* | 100 | 100 | 100 | 100 | 100 | 100 |
| *Candida glabrata* | 61 | 49 | 75 | 53 | 57 | 59 |
| *Candida kefyr* | 56 | 59 | 68 | 55 | 53 | 58 |
| *Candida albicans* | 46 | 38 | 62 | 42 | N/A | 47 |
| *Candida parapsilosis* | 46 | 34 | 65 | 43 | 30 | 44 |
| *Candida guilliermondii* | 44 | 30 | 64 | 46 | 31 | 43 |
| *Candida lusitaniae* | 43 | 34 | 67 | 44 | 26 | 43 |
| *Candida auris* | 43 | 35 | 63 | 45 | 25 | 42 |
| *Candida krusei* | 38 | 35 | 61 | 39 | 25 | 40 |
| *Homo sapiens* | 29 | 28 | 46 | 32 | 22 | 31 |

Sequence identities (%) and subunit names are based on the homologs of *Saccharomyces cerevisiae*, sorted in descending order of average identity. N/A denotes the absence of a known homolog.
